# Adaptor-Specific Antibody Fragment Inhibitors for the Intracellular Modulation of p97 (VCP) Protein–Protein Interactions

**DOI:** 10.1021/jacs.2c03665

**Published:** 2022-07-12

**Authors:** Ziwen Jiang, Yu-Hsuan Kuo, Mengqi Zhong, Jianchao Zhang, Xin X. Zhou, Lijuan Xing, James A. Wells, Yanzhuang Wang, Michelle R. Arkin

**Affiliations:** † Department of Pharmaceutical Chemistry, University of California, San Francisco, California 94158, United States; ‡ Small Molecule Discovery Center, University of California, San Francisco, California 94158, United States; § Department of Molecular, Cellular and Developmental Biology, University of Michigan, Ann Arbor, Michigan 48109-1085, United States; ∥ Department of Cancer Biology, Dana-Farber Cancer Institute, Boston, Massachusetts 02215, United States; ⊥ Department of Biological Chemistry and Molecular Pharmacology, Harvard Medical School, Boston, Massachusetts 02115 United States

## Abstract

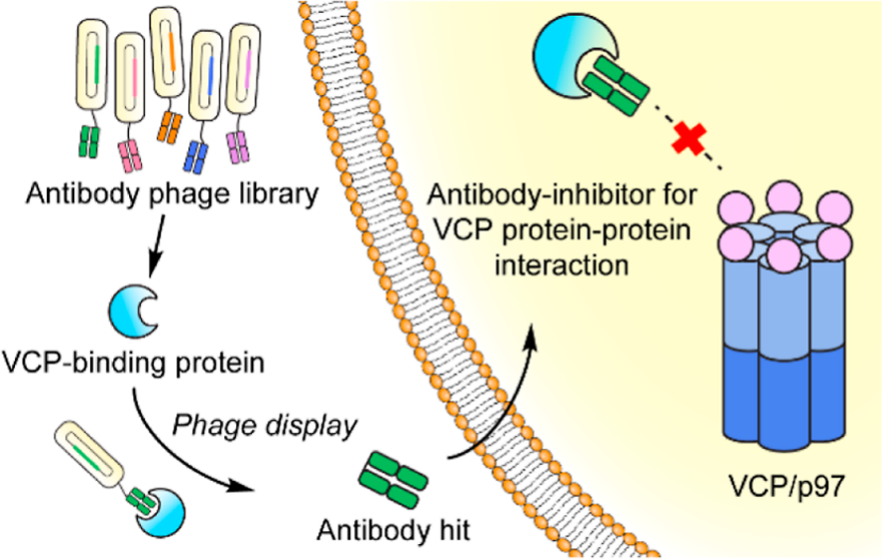

Protein–protein interactions (PPIs) form complex networks to drive cellular signaling and cellular functions. Precise modulation of a target PPI helps explain the role of the PPI in cellular events and possesses therapeutic potential. For example, valosin-containing protein (VCP/p97) is a hub protein that interacts with more than 30 adaptor proteins involved in various cellular functions. However, the role of each p97 PPI during the relevant cellular event is underexplored. The development of small-molecule PPI modulators remains challenging due to a lack of grooves and pockets in the relatively large PPI interface and the fact that a common binding groove in p97 binds to multiple adaptors. Here, we report an antibody fragment-based modulator for the PPI between p97 and its adaptor protein NSFL1C (p47). We engineered these antibody modulators by phage display against the p97-interacting domain of p47 and minimizing binding to other p97 adaptors. The selected antibody fragment modulators specifically disrupt the intracellular p97/p47 interaction. The potential of this antibody platform to develop PPI inhibitors in therapeutic applications was demonstrated through the inhibition of Golgi reassembly, which requires the p97/p47 interaction. This study presents a unique approach to modulate specific intracellular PPIs using engineered antibody fragments, demonstrating a method to dissect the function of a PPI within a convoluted PPI network.

## Introduction

Protein–protein interactions (PPIs) are essential for intracellular signal transduction and transcriptional regulation.^1,2^ These cellular events are generally controlled by networks that are composed of several interconnected PPIs. Misregulation of these cooperative PPIs has been shown to cause diseases such as cancer and neurodegeneration.^3,4^ Aberrant PPIs include either the loss of a crucial interaction or the gain of a spatiotemporally incorrect interaction.^5^ The advancement of proteomics has facilitated the understanding of PPI networks, specifically in elucidating the interacting protein partners.^6,7^ Precisely mapped PPI networks provide fundamental information to explore the possibilities in controlling cellular functions through specific modulation of protein complexes. Therefore, we seek systematic methodologies for PPI inhibition or stabilization to understand the function of PPI and select the targets with relevant therapeutic avenues for disease treatment.^8,9^

An ideal PPI-specific modulator tool should focus on the interaction between the protein partners of interest, leaving other functions and PPIs of the targeted protein partners unaltered. Efficient genetic modulation methods such as knockdown^10^ or knockout assays^11^ do not offer such PPI-specific regulation as the depletion of one protein will remove all of its PPIs simultaneously. Likewise, overexpression of the protein of interest can lead to multiple enhanced PPIs, cumulatively affecting the network. Therefore, the development of PPI-specific modulation can benefit from selective blockade of the PPI interface. Extracellular proteins are readily orthosterically inhibited by antibodies, whereas small molecules and peptides are most often targeted to intracellular PPIs. However, a lack of pockets or grooves and the relatively large area of the PPI interfaces pose challenges for discovering small-molecule modulators, making it difficult to investigate multiple related PPIs rapidly and systematically.^1,12−14^

Antibodies possess unique properties as potential intracellular PPI regulators when compared to small molecules and peptides. The protein nature of antibodies allows convenient cloning modifications to install subcellular localization signals,^15,16^ precisely refining the intracellular function of these antibodies to the targeted cellular milieu. The variable platforms of antibodies are available from nanobodies (∼15 kDa),^17^ single-chain variable fragments (scFvs, ∼27 kDa),^18^ and antigen-binding fragments (Fabs, ∼50 kDa)^19^ to full-length IgG (∼150 kDa), offering a tunable size range to tackle different PPI interfaces. Moreover, the constant chain within these antibody platforms enhances their stability against hydrolysis.^20,21^ Nevertheless, antibody-based modulators so far are mainly applied to secreted proteins or cell membrane targets.^22,23^

**Figure 1:**
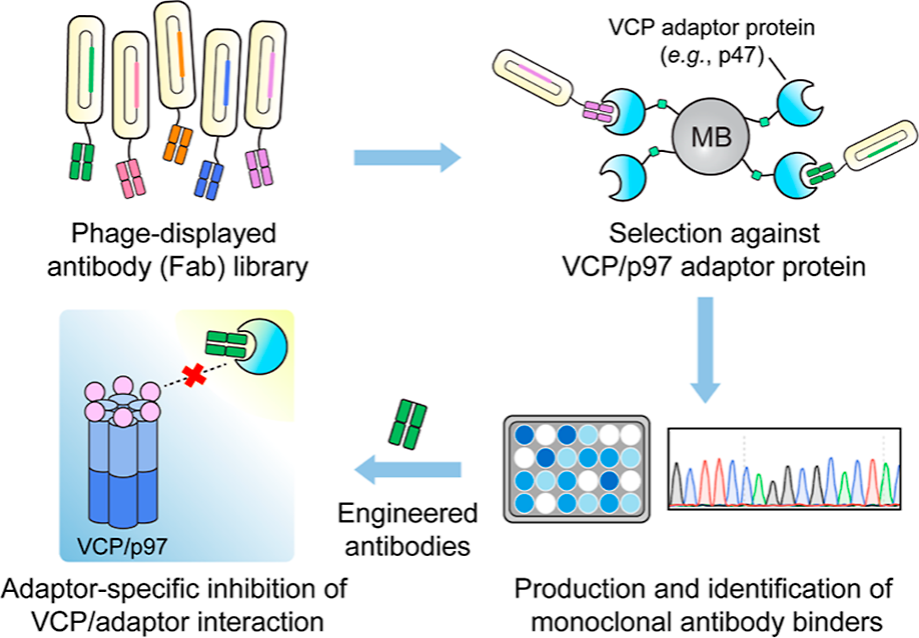
Workflow for the discovery of antibody modulators for specific VCP PPIs through phage display. MB, magnetic beads.

As an intracellular hub protein, valosin-containing protein (VCP/p97) interacts with more than 30 adaptor proteins to regulate multiple cellular functions, including the maintenance of protein homeostasis and facilitating protein degradation.^24−26^ Dissecting the particular function of an individual p97/adaptor protein interaction is therefore important but complicated within the p97 PPI network. In this work, we engineered antibody fragment inhibitors via phage display to modulate the interaction between p97 and its adaptor protein, NSFL1C (p47) (Figure 1). We chose the p97/p47 interaction as our model target for the development of PPI-specific antibody-based modulators because it is involved in membrane fusion processes,^27^ particularly during Golgi fragmentation and reassembly that are distinctive and readily measured.^28^ These engineered anti-p47 antibody fragments with nanomolar binding affinities successfully disrupted the intracellular interaction between p97 and p47. Expressing variations of the antibody fragments and nuclear localization signal sequences resulted in different phenotypic responses for Golgi fragmentation, further elucidating the role of p97/p47 interaction during Golgi dynamics. The study highlights a unique antibody-based approach for intracellular modulation of the p97/p47 interaction, providing a new tool to untangle the convoluted PPI networks during a cellular process.

## Results and Discussion

### Engineered Antibody Fragments for the p47-UBX Domain Demonstrated Nanomolar Binding Affinities and High Selectivity

**Figure 2:**
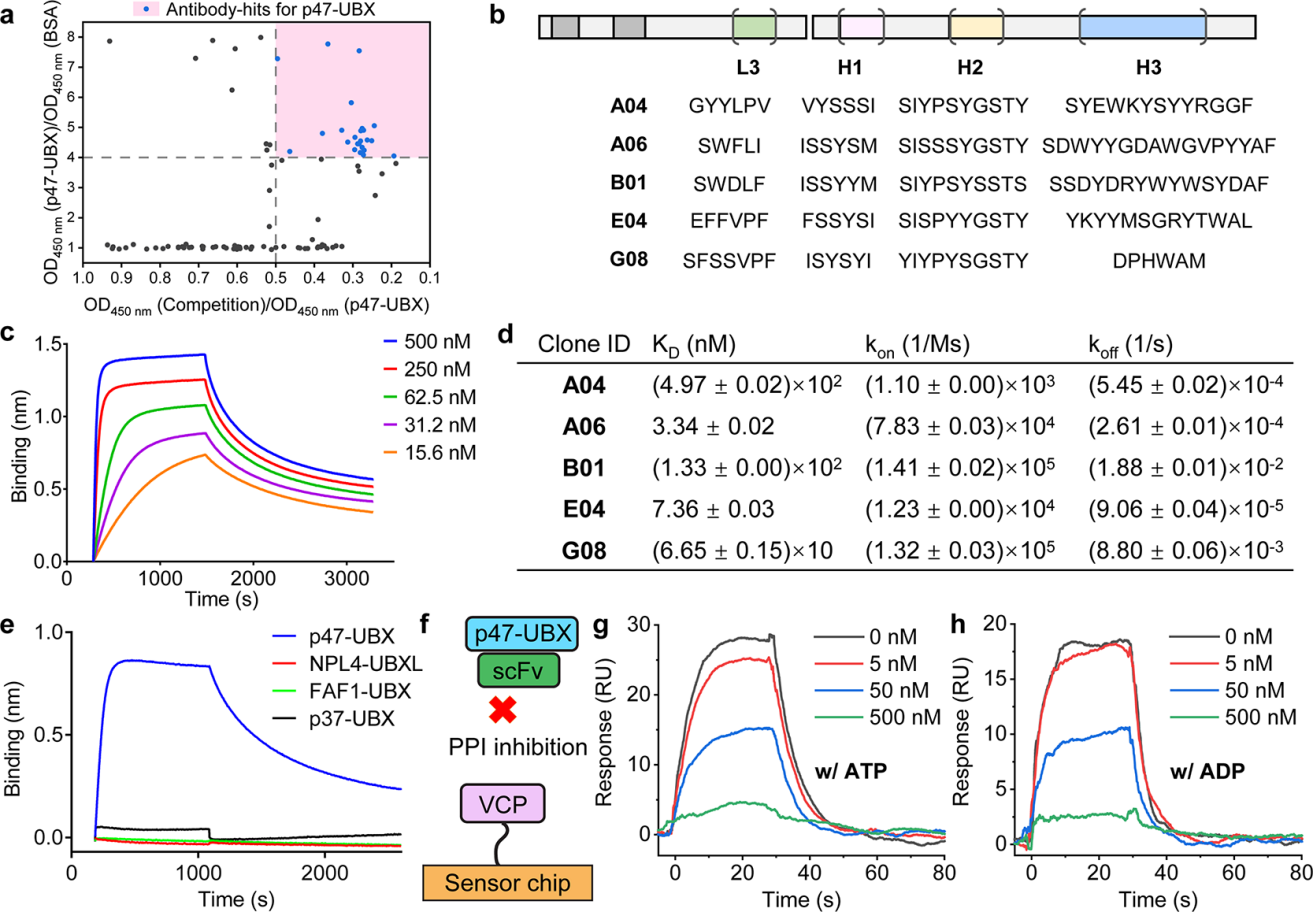
Selection and characterization of anti-p47-UBX antibody fragments. (a) Plot of phage ELISA to select the binders for the p47-UBX domain. The ratio of OD_450nm_ (p47-UBX)/OD_450nm_ (BSA) represents the selectivity of the binder, where a higher ratio represents a more specific binder. The ratio of OD_450nm_ (competition)/OD_450nm_ (p47-UBX) represents the capability of the soluble p47-UBX to compete with the p47-UBX coated on the plate for Fab-phage binding, where a lower ratio value generally indicates a tighter binder. The pink quadrant represents the hit set. (b) Sequence of the CDRs for selected antibody binders to p47-UBX. (c) BLI dose-response profiles of scFv-A06 to the p47-UBX domain. (d) Binding affinities of selected scFvs for p47-UBX based on BLI results. Standard deviations represent *N* = 2 independent experiments. (e) BLI results of scFv-A06 binding to the interacting domains of different p97 adaptor proteins, showing selectivity for p47 over NPL4, FAF1, and p37. (f) Schematic illustration of the SPR assessment of the competition between selected scFv and p47-UBX for p97 binding. (g,h) SPR sensorgrams for the scFv-A06/p47-UBX mixture binding to full-length human p97 in the presence of either 100 μM ATP (g) or 100 μM ADP (h) at the listed concentrations of scFv. Data are representative of *N* = 2 independent experiments.

We chose the UBX (ubiquitin regulatory X) domain of p47 as the antigen to discover inhibitors of p47/p97 by Fab phage display. Previous studies have shown that the p97-N terminal domain directly interacts with the p47-UBX domain, and we hypothesized that some anti-p47-UBX antibodies would inhibit the p47/p97 interaction.^29^ Furthermore, antibodies against p97 would likely result in the inhibition of multiple PPIs because most adaptors bind to a common site on the p97-N domain; 13 different p97 adaptors contain a UBX domain.^30,31^ Thus, inhibition from the p47 side should be more specific. To engineer antibodies for the p47-UBX domain, we carried out phage display using a previously developed Fab phage library.^32^ The Fab scaffold derived from the 4D5 anti-HER2 antibody is highly stable (T_m_ 80 °C) and designed with amino acid variations in four of the six complementarity-determining regions (CDRs).^32,33^ After four rounds of selection against the p47-UBX domain, more than five clones were identified through phage ELISA (Figures 2a and S1). These selected Fab hits (Figure 2b) for p47-UBX were sequenced and cloned from a phagemid into scFv platforms for monoclonal antibody production.

Next, we assessed the binding affinity between the anti-p47-UBX scFv and p47-UBX domain using biolayer interferometry (BLI). After anchoring biotinylated p47-UBX on the BLI sensor tip, an increasing amount of each scFv was separately incubated with the p47-UBX-decorated sensor. From the dose response profiles, scFv-A06 and scFv-E04 demonstrated nanomolar binding affinities (∼3 and ∼7 nM, respectively) to the p47-UBX domain (Figures 2c,d and S2). We also evaluated the selectivity of these scFvs against the p97-binding domain of other adaptor proteins [*e.g.*, NPL4 (nuclear protein localization homologue 4), FAF1 (FAS-associated factor 1), and p37 (UBX domain protein 2B)] using BLI. All five scFv clones were highly selective toward the p47-UBX domain when compared to the NPL4-UBXL and FAF1-UBX domain (Figures 2e and S3). Moreover, as p47 and p37 have overlapping functions and are highly homologous (65% sequence identity; Figure S4),^34^ it was critical to ensure that the selected anti-p47-UBX scFv did not bind to the p37-UBX domain. Upon evaluating 2 anti-p47-UBX scFv nanomolar binders with BLI, scFv-E04 did bind to p37-UBX with a dissociation constant (*K*_D_) of ∼4 nM, while scFv-A06 did not bind to p37-UBX (Figure S5). Overall, scFv-A06 is the most selective clone for the p47-UBX domain with a *K*_D_ of ∼3 nM.

### Anti-p47-UBX Antibody Fragments Disrupt the p97/p47 PPI *In Vitro*

Although selected based on binding to the p47-UBX domain, an essential requirement for these anti-p47-UBX antibody fragments is that they can disrupt the PPI of interest. We employed surface plasmon resonance (SPR) to test if these scFvs interfered with the p97/p47 interaction. As a flow-based approach, SPR offers a dynamic monitoring of the PPI status.^35,36^ We anchored biotinylated full-length p97 on a streptavidin-functionalized SPR sensor chip, followed by flowing a mixture of increasing concentrations of scFvs and a fixed concentration of p47-UBX. Considering the conformational changes of full-length p97 in the presence of either ATP or ADP,^37^ we conducted the SPR evaluation with both nucleotides. By SPR, we observed that four selected scFvs (A06, B01, E04, and G08) disrupted the p97/p47-UBX interaction, regardless of the presence of nucleotide (Figures 2f–h and S6). In the presence of an equal molar amount of p47-UBX and scFv-A06 (both at 50 nM), the binding of p47-UBX to the p97-anchored sensor was reduced ∼50% when compared with the control group where scFv-A06 was not present. Recent data indicated that there are three p97-binding modules on p47.^38^ Our construct for phage display included the C-terminal SHP motif and UBX domain but lacked the N-terminal SHP motif of p47 (Figure S7a). We therefore evaluated the full-length p47 and found that the same scFvs that inhibited the p97/p47-UBX interaction also blocked the p97/full-length p47 interaction (Figure S7), demonstrating that our p47 construct for phage display was adequate for selecting inhibitors of the full-length p47.

**Figure 3:**
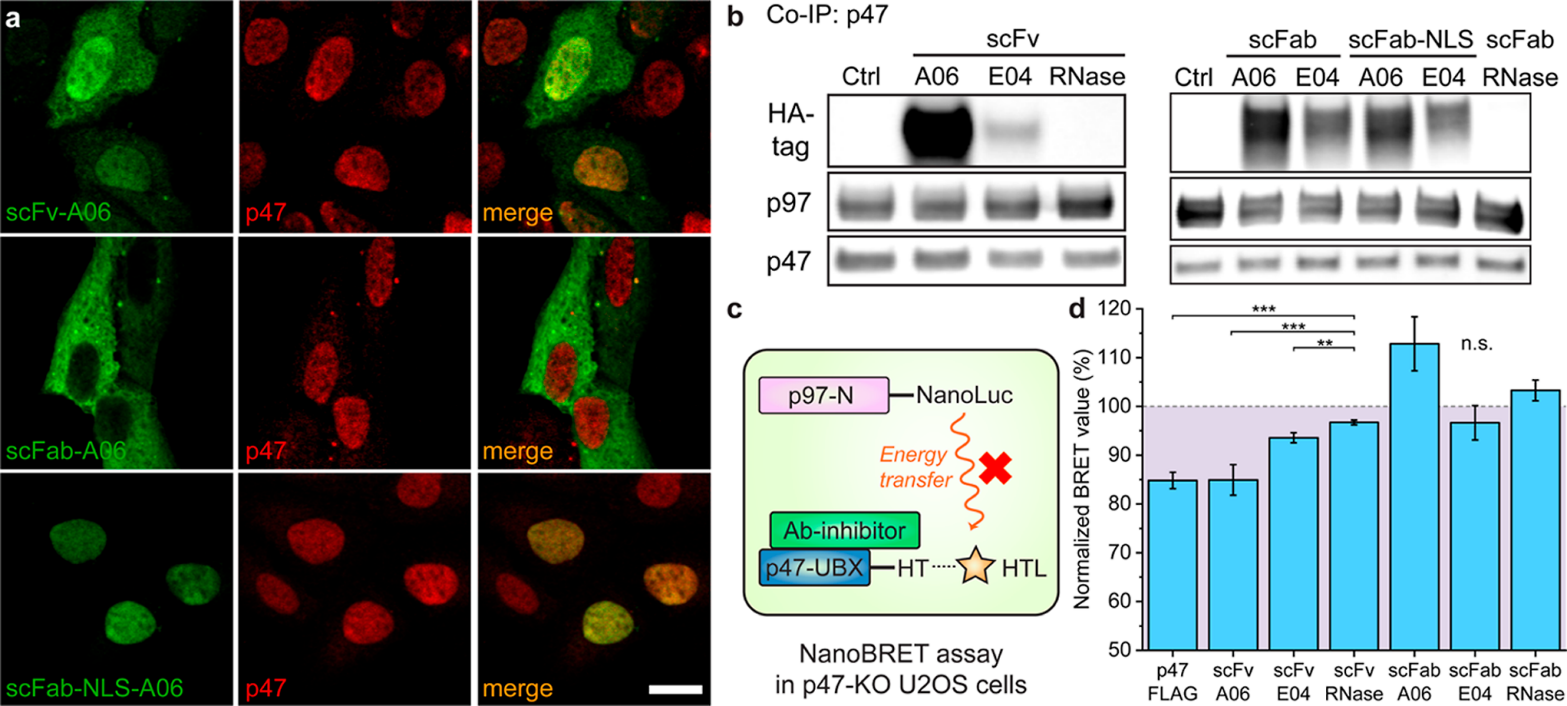
Anti-p47-UBX antibody fragments interact with p47 and inhibit p97 binding in U2OS cells. (a) Representative images for the cellular localization of p47 and selected antibody fragments in U2OS cells. Plasmids that encode antibody fragments were transfected in U2OS cells for 24 h. Scale bar, 20 μm. (b) Co-IP of p47 from U2OS cells after transfection of plasmids that encode antibody fragments. P47-containing protein complexes were captured from the lysates and blotted for the co-IP analysis. Data represent *N* = 2 independent experiments. (c) Schematic illustration of the NanoBRET assay for p97/p47 interaction in the presence of antibody fragment inhibitors (Ab-inhibitor). NanoLuc, nanoluciferase; HT, HaloTag; HTL, HaloTag ligands. (d) NanoBRET assay in p47-knockout (p47-KO) U2OS cells. ScFv-A06 reduces the p97-N/p47-UBX interaction signal as well as the p47-overexpression group does. The dashed line represents the normalized BRET ratio of the average between scFv-RNase and scFab-RNase groups. Error bars represent standard deviations of *N* = 4. Statistical analyses are performed using two-tailed Student’s *t*-test. **p < 0.01; ***p < 0.001; and n.s., no significance.

Translating these antibodies into functional PPI modulators requires their intracellular presence. Therefore, we cloned the encoding sequences of these antibody fragments into a mammalian expression vector. In the mammalian expression vector design, we included both the scFv and scFab (single-chain Fab fragment) formats and the N-terminal nuclear localization signal (NLS)-tagged^39^ scFab (scFab-NLS) format in order to vary their cellular localization. Each clone contained a C-terminal HA (human influenza hemagglutinin) epitope tag for detection purposes. After transfecting these plasmids with Xfect transfection reagents for 24 h in human bone osteosarcoma epithelial (U2OS) cells (Figure S8), we visualized the antibody fragments and p47 by immunofluorescence (IF) (Figure 3a). P47 primarily localized in the nucleus, agreeing with previous reports.^28^ ScFv-A06 showed some cytoplasmic distribution but primarily colocalized with p47 in the nucleus, suggesting the interaction between scFv-A06 and p47. Interestingly, due to the passive diffusion limit for the nuclear pore (∼60 kDa),^40,41^ scFab (∼60 kDa) primarily localized in the cytosol, whereas scFab-NLS was mostly confined to the nucleus and overlapped with p47. Due to the low intensity of cytoplasmic p47, it was difficult to confidently state that scFab-A06 colocalized with p47 in the cytosol. Taken together, IF results indicated that the molecular weight of the antibody fragments and the nuclear localization tag determined the localization of the antibody fragment. Western blotting of the scFab and scFv antibody fragments indicated that E04 was expressed less than A06 antibody fragments in U2OS cells, possibly due to a lack of stability (Figure S9).

We also confirmed the p47/antibody fragment interaction through co-immunoprecipitation (co-IP) of p47. All A06- and E04-based antibody fragments (scFv, scFab, and scFab-NLS) bound to p47 (Figure 3b). Binding of scFab in the lysates demonstrated that this format was capable of binding even though it was not colocalized in intact cells. Interestingly, we observed that p47 was not completely detached from p97 upon the binding of antibody fragments. We next evaluated the co-IP of p97 and confirmed the formation of the trimeric complex containing p97, p47, and scFv-A06 (Figure S10). This result contrasted with our biophysical experiments, where these antibody fragments could completely compete with full-length p97 (Figures 2g,h and S6, S7). The cellular result was understandable for a few reasons. First, endogenous levels of protein expression were not precisely controlled, in contrast to our biophysical assays using recombinant proteins; hence, the expression level of scFv-A06 when compared to that of p47 might have been insufficient to completely displace p47 from p97. Second, as previous studies have shown that p47 has an oligomeric form,^42^ one p47 “arm” might have been inhibited by antibody fragments while the other(s) remained bound. Third, a recent report demonstrated that the UBX domain is not the only module of p47 that binds to p97.^38^ Our antibody fragments might have blocked only the UBX domain and/or the C-terminal SHP motif of p47 that were present in the selection construct (Figure S7a) during phage display, while the N-terminal SHP motif of p47 retained its interaction with the adjacent p97 protomer.

We next utilized NanoBRET (bioluminescence resonance energy transfer) assay^43^ to quantitatively probe the effect of engineered antibody fragments on the p97/p47 interaction. The optimized NanoBRET assay for p97/p47 PPI contained an N-terminal nanoluciferase-tagged p97-N domain (pNLF1N-p97-N) as the donor and a C-terminal Halo-tagged p47-UBX domain (pHTC-p47-UBX) as the acceptor (Figure 3c). To rule out the potential competition with the NanoBRET pair by endogenous p47, we generated p47-knockout (p47-KO) U2OS cells by CRISPR/Cas9 genome editing (Figure S11). During the NanoBRET evaluation, the donor, acceptor, and antibody fragment (scFv and scFab)-expressing plasmids were co-transfected into the p47-KO U2OS cells to evaluate the proximity change between the NanoBRET pair (Figure S12). In parallel, we employed the scFv and scFab platforms of the anti-RNase (ribonuclease A) antibody^44^ as negative controls and the p47-expressing vector (p47-FLAG) as the positive control. When compared to the BRET ratio in the scFv-RNase and scFab-RNase control groups, the co-transfection of either scFv-A06 or p47-FLAG similarly reduced the BRET ratio by ∼15%, indicating a reduced interaction between the p97/p47 NanoBRET pair (Figures 3d and S12c). In comparison, the lack of effect on the BRET signal from scFv-E04 may be attributed to its low expression level (Figure 3b). The lack of effect of scFab-A06 on the BRET signal may be attributed to the localization of the antibody fragment in the cytosol (Figure 3a). Based on the colocalization, co-IP, and NanoBRET results, scFv-A06 was the most potent inhibitor of the p97/p47 PPI among the anti-p47-UBX antibody fragments tested.

### Antibody Fragments Inhibit Reassembly of the Golgi Apparatus

**Figure 4:**
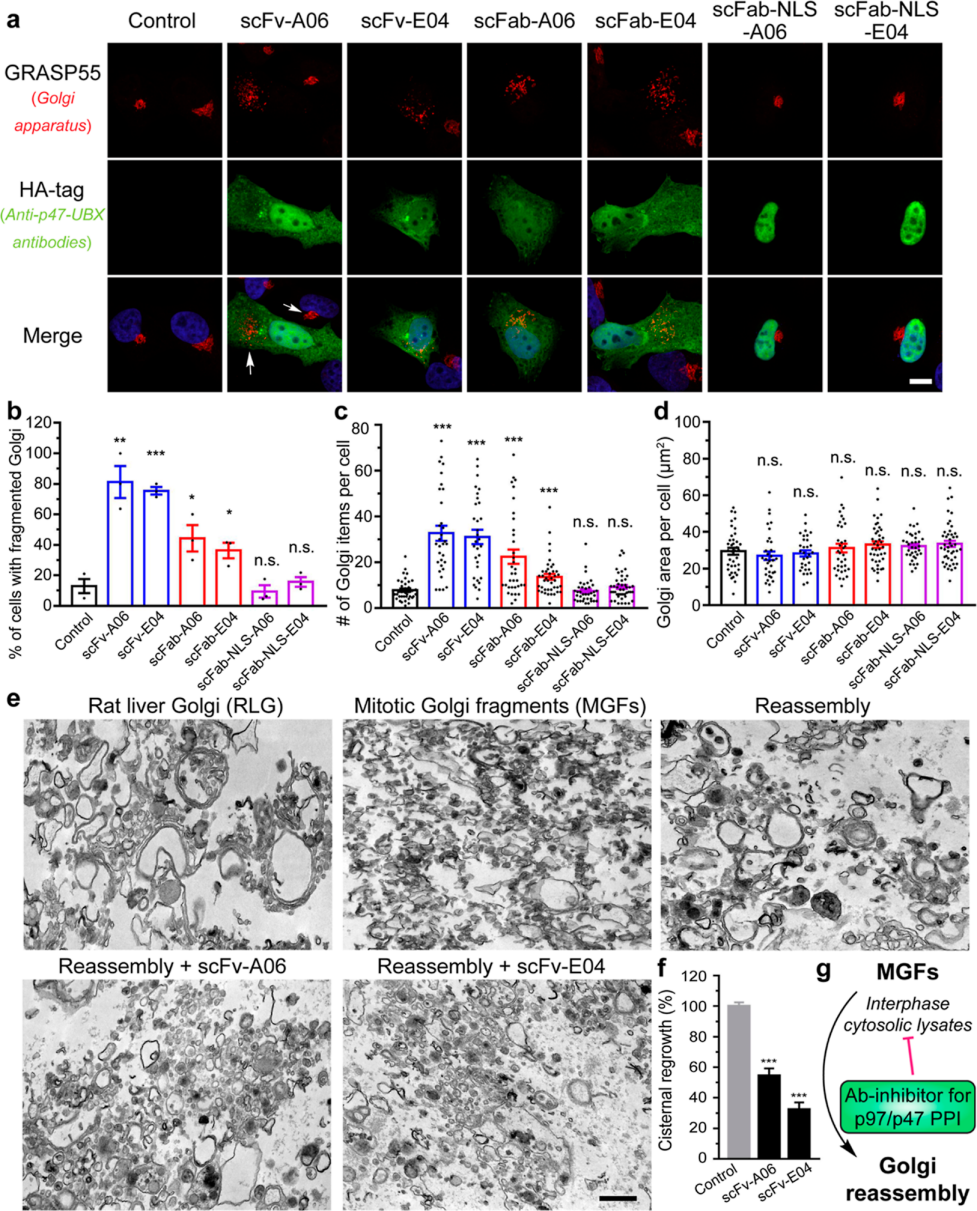
Anti-p47-UBX antibody fragments disrupt the Golgi structure by inhibiting its post-mitotic reassembly process. (a) Representative immunofluorescence images of HeLa cells transfected with HA-tagged anti-p47-UBX antibody fragments for 24 h and stained with an antibody to Golgi marker GRASP55. Scale bar, 10 μm. (b–d) Quantification of GRASP55 for the percentage of cells with fragmented Golgi (b), number of Golgi items per cell (c), and the Golgi area per cell (d). Data are shown as mean ± SEM from *N* = 3 independent experiments. (e) Representative transmission electron microscopy images of RLG, MGF (RLG treated with mitotic cytosol), and reassembled samples (MGF treated with interphase cytosol). In brief, RLG membranes were fragmented by treatment with mitotic HeLa cytosol, and MGFs were reisolated and incubated with interphase cytosol alone or in the presence of recombinant anti-p47-UBX scFvs. Scale bar, 500 nm. (f) Quantification of the cisternal regrowth in (e). Results are shown as the mean percentage of membranes in cisternae ±SEM, where 0% represents cisternal regrowth in MGF (10.8 ± 1.7% of membranes in cisternae) and 100% represents cisternal regrowth of MGFs incubated with interphase cytosol alone (56.7 ± 1.1% of membranes in cisternae). Statistical analyses were performed using two-tailed Student’s *t*-test. *p < 0.05; **p < 0.01; ***p < 0.001; and n.s., no significance. (g) Scheme showing antibody fragment inhibitors of p97/p47 PPI-inhibiting Golgi reassembly.

Antibody fragment-based PPI inhibitors are expected to affect the PPI-related phenotypes and modulate cellular functions. The p97/p47 complex facilitates the fusion of Golgi membrane fragments to reassemble the Golgi apparatus during the cell cycle at the late mitosis and early interphase.^28^ Therefore, we evaluated the effect of A06 and E04 anti-p47-UBX antibody fragments on Golgi reassembly processes using the morphological distribution of GRASP55, a Golgi reassembly stacking protein that reflects the Golgi structure.^45^ After transfecting the antibody fragment-expressing plasmids into HeLa cells, the two scFv and scFab clones significantly increased Golgi fragmentation, with the scFv groups showing more Golgi fragments per cell than the scFab groups (Figures 4a–c and S13). Since the Golgi area per cell remained constant (Figure 4d), an increased number of Golgi fragments indicated an attenuated reassembly process of Golgi membranes. Both p47 and the close homologue p37 have been associated with Golgi reassembly.^34^ It is noteworthy that A06 was selective for p47, whereas E04 bound similarly to both p47 and p37 (Figure S4). The dual-inhibition mechanism of E04 may have accounted for the effective inhibition of Golgi reassembly, even given the lower expression level of E04 compared to that of A06.

The tunability of the antibody fragment platform contributed to the understanding of p97/p47 PPI in Golgi reassembly. Previous mechanistic studies revealed that the p97/p47 complexes participated in membrane fusion during mitosis, whereas p47 primarily resided in the nucleus during the interphase in HeLa cells.^28^ It was therefore unexpected that expression of both the scFvs and scFabs without an NLS tag yielded fragmented Golgi, while the scFabs with NLS tags showed minimal interference on the Golgi morphology. The cytoplasmic distribution of antibody fragments was therefore correlated with activity, suggesting the importance of p47 during phases of the cell cycle where the nuclear membrane was intact.

To further validate the effect of these scFvs on Golgi reassembly, we carried out a cell-free Golgi reassembly assay^29,46,47^ using purified rat liver Golgi (RLG) membranes. Our engineered scFvs for human p47 also bound to rat p47 (Figure S14). In the *ex vivo* assay, RLG membranes were treated with mitotic cytosolic lysates of HeLa cells to form mitotic Golgi fragments (MGFs), that is, disassembling the Golgi cisternae into smaller vesicles (Figure 4e). Next, the MGFs were treated with interphase cytosolic lysates from HeLa cells that contained endogenously expressed p97/p47 complexes, inducing reassembly of the MGFs to large Golgi cisternae.^46^ When scFv-A06 or scFv-E04 was spiked into the interphase cytosolic lysates, the cisternal regrowth was slowed down to less than 50% (Figure 4f), confirming the inhibitory effect of these engineered antibody fragments as PPI modulators during Golgi reassembly (Figure 4g).

## Conclusions

We have described the engineering of antibody fragment inhibitors for a specific PPI of the multifunctional protein p97 to modulate a cellular function of interest. These antibody fragments were selected by Fab phage display for binding to the p97-interacting domain of the adaptor protein p47. Compared to genetic knockdown and knockout approaches, the well-defined steric blockade by these antibody inhibitors potentially reduces the interference to the overall p97 PPI network. Such antibody fragments are available in tunable molecular weights and subcellular localization tags through protein engineering, making it an attractive approach to study the PPI of interest at different cellular locations and providing toolkits to tackle biological questions. For example, this work demonstrates the first reported antibody fragment that modulates the Golgi structure. As Golgi defects are observed in an increasing number of human diseases,^48,49^ this antibody fragment inhibitor could potentially be useful in aiding the development of disease treatment.

Although we and others have worked to develop small-molecule inhibitors of PPI,^50,51^ it is still challenging to develop inhibitors for highly related targets systematically. By contrast, phage-selected antibody fragments can readily be developed for a panel of PPIs with a common hub protein. Furthermore, antibody discovery by phage display can also be applied to protein targets with post-translational modifications, protein isoforms, splice variants, or conformational variants, tremendously improving the applicability of our antibody inhibitor platform. Currently, the cell membrane and endosomal entrapment of proteins limit the ability to deliver antibodies intracellularly.^52^ Intracellular antibodies have been used as PPI modulators in a few cases, such as the signaling pathway of RAS proteins, and we now show their utility for selectively blocking one PPI of a hub protein that binds to many proteins at the same site.^53−55^ Development of efficient and robust methods for delivering recombinant antibodies^56^ to intracellular PPI targets will further extend the applicability of the technology for biomedical applications, ideally by perturbing the formation of malfunctional protein complexes that are associated with diseases and disorders.

## Supporting Information Available

Supplementary figures, methods, sequences, spectral, and characterization data (PDF)
