# Supplementary material for: Adaptor-Specific Antibody Fragment Inhibitors for the Intracellular Modulation of p97 (VCP) Protein–Protein Interactions
Source: J Am Chem Soc. 2022 Jul 12;144(29):13218–25. doi: 10.1021/jacs.2c03665 (PMC9335864; doi:10.1021/jacs.2c03665)
Supplement: Supplementary file 1 — ja2c03665_si_001.pdf [file ja2c03665_si_001.pdf]

Supporting Information

Adaptor-specific antibody fragment inhibitors for the intracellular modulation of p97 (VCP) protein-protein interactions

Ziwen Jiang,[†,‡,§] *Yu-Hsuan Kuo,*[†,‡,§] *Mengqi Zhong,*[†,‡,§] *Jianchao Zhang,*[¶,§] *Xin X. Zhou,*[†,#,||] *Lijuan Xing,*[¶]

James A. Wells,[†] *Yanzhuang Wang,*[¶] *Michelle R. Arkin*[*,†,‡]

[†]Department of Pharmaceutical Chemistry, [‡]Small Molecule Discovery Center, University of California, San Francisco, CA 94158, USA, [¶]Department of Molecular, Cellular and Developmental Biology, University of Michigan, Ann Arbor, MI 48109-1085, USA, [#]Department of Cancer Biology, Dana-Farber Cancer Institute, Boston, MA 02215 USA, [||]Department of Biological Chemistry and Molecular Pharmacology, Harvard Medical School, Boston, MA 02115 USA.

[§]These authors equally contributed to this work.

[*]Email: michelle.arkin@ucsf.edu (M.R.A.)

Table of Contents

1. Supplementary figures

Figure S1. Selection process with Fab-phage libraries.

Figure S2. Biolayer interferometry (BLI) dose response profiles of scFv binders to p47-UBX domain.

Figure S3. BLI results of selected scFv binders to the interacting domains of p97 adaptor proteins.

Figure S4. Amino acid sequence alignment between p37-UBX and p47-UBX and their key binding residues with p97-N domain.

Figure S5. BLI dose response profiles of scFv-A06 and scFv-E04 to p37-UBX domain.

Figure S6. Surface plasmon resonance (SPR) sensorgrams showing the competition between scFvs and p47-UBX for binding with p97.

Figure S7. Surface plasmon resonance (SPR) sensorgrams showing the competition between scFvs and p47 (or p47-UBX) for binding with p97.

Figure S8. Flow cytometry histograms of U2OS cells transfected with EGFP-expressing plasmids using Xfect.

Figure S9. Western blots for HA-tag, p47, p97, and vinculin in U2OS cells after 24-h transfection of antibody fragments.

Figure S10. Co-immunoprecipitation of p97 and western blot results in U2OS cells after scFv transfection.

Figure S11. Generation of p47-knockout (p47-KO) U2OS cells.

Figure S12. Optimization of the NanoBRET assay for probing the p97/p47 interaction.

Figure S13. Representative immunofluorescence images of a Golgi marker GRASP55 in HeLa cells transfected with anti-p47-UBX antibody fragments.

Figure S14. Co-immunoprecipitation and western blot results in HeLa, Rat-1, and NRK cells after scFab-NLS transfection.

2. Supplementary methods

2.1. Plasmid construction

2.2. Protein expression and purification

2.3. Fab-phage selection

2.4. Phage ELISA

2.5. Biophysical characterizations

2.6. Cell culture

2.7. Immunofluorescence (IF)

2.8. Co-immunoprecipitation (Co-IP) and western blot

2.9. NanoBRET p97/p47 PPI assay

2.10. Quantitative Golgi assays

3. Supplementary sequences

4. Spectral and characterization data

5. References

1. Supplementary Figures and Table

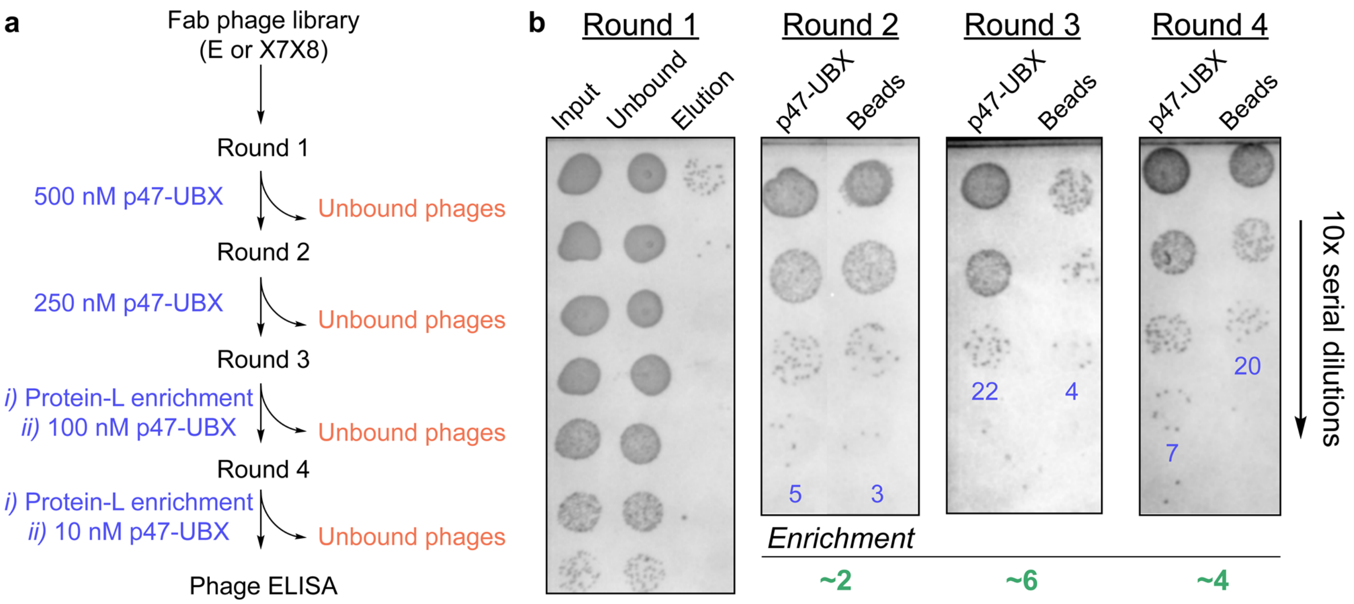

Figure S1. Selection process with Fab-phage libraries. (a) Workflow of phage display to isolate binders to p47-UBX domain from Fab-phage libraries. (b) Titers of Fab-phage after 4 rounds of selection. Round 2~4 demonstrated enrichment for Fab-phage binders to p47-UBX over SA-functionalized magnetic beads.

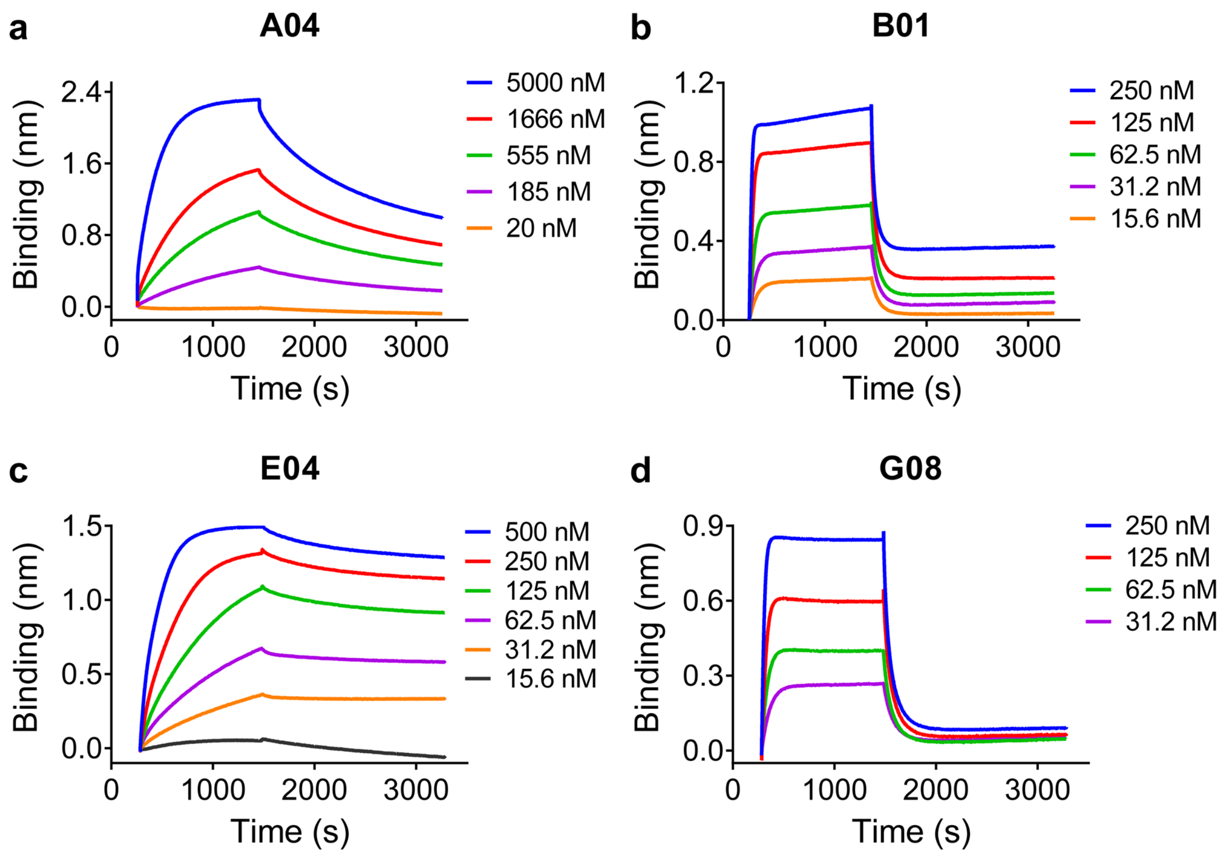

Figure S2. Biolayer interferometry (BLI) dose response profiles of scFv binders to p47-UBX domain. Data represent $N = 2$ independent experiments.

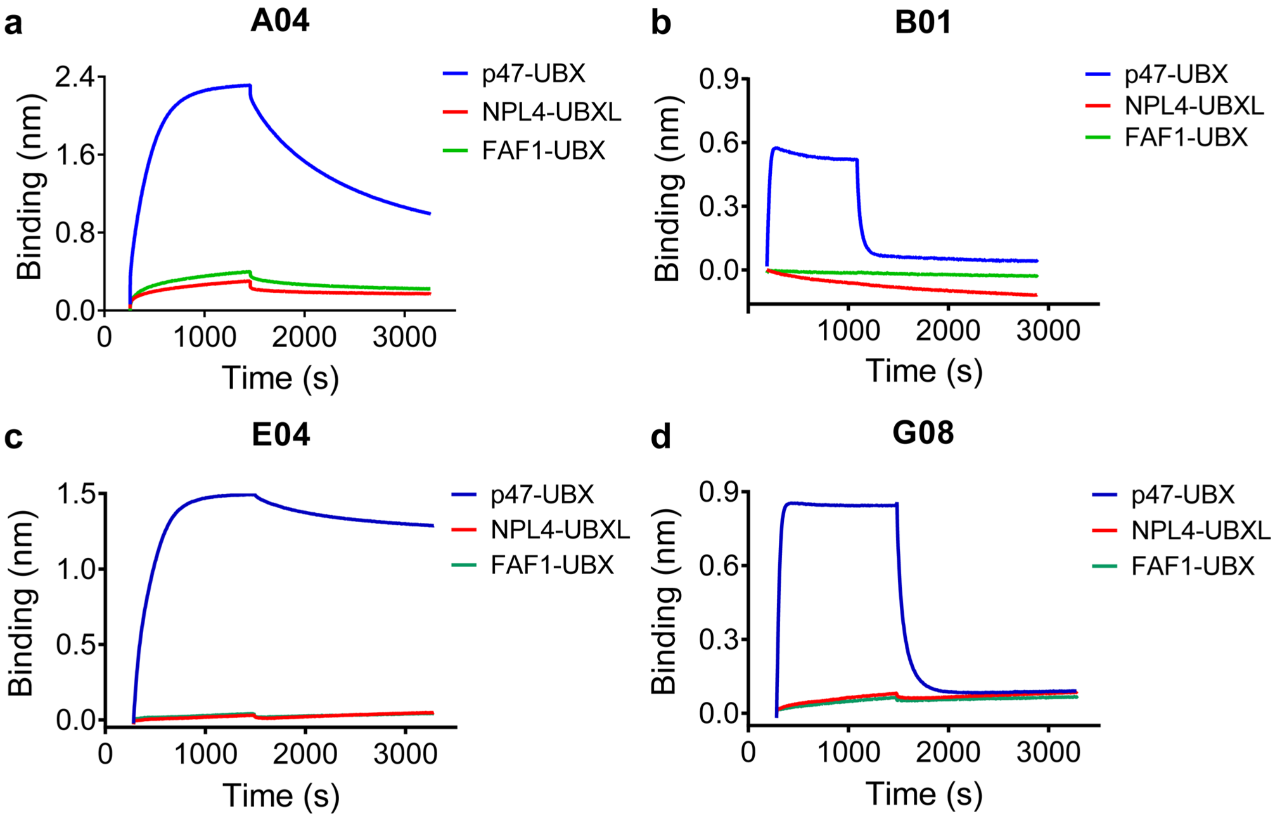

Figure S3. BLI results of selected scFv binders to the interacting domains of p97 adaptor proteins. Data represent $N = 2$ independent experiments.

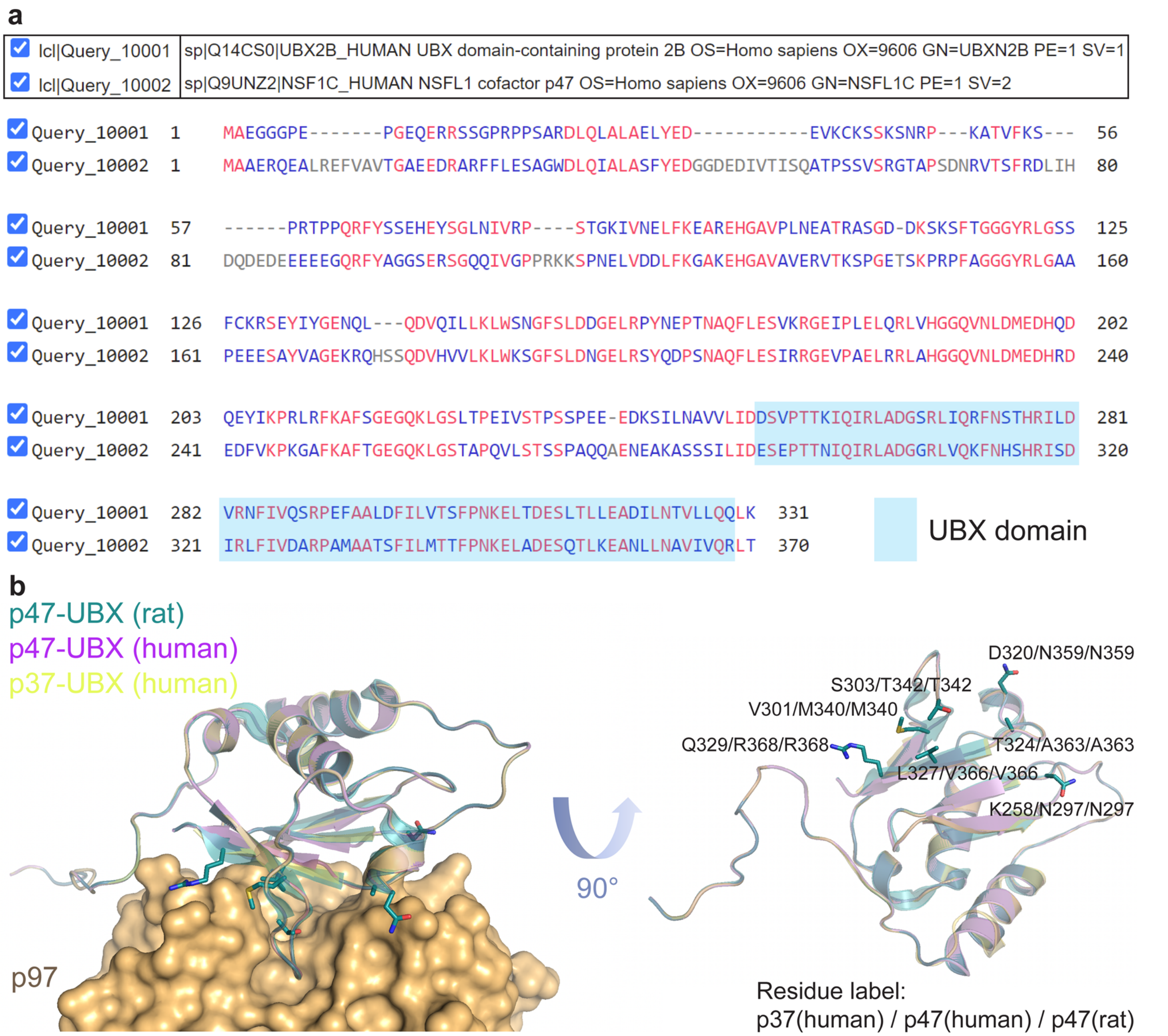

Figure S4. (a) Amino acid sequence alignment of human p37 (UBX2B) and p47 (NSFL1C) using the NIH COBALT alignment tool. The p47-UBX domain is highly homologous to the p37-UBX domain (~64% sequence identity). (b) Structure of a p97-N domain (shown in brown) with the associated rat p47-UBX domain (shown in cyan; reported crystal structure[SR1]), human p47-UBX domain (shown in purple; generated with SWISS-MODEL), or p37-UBX domain (shown in yellow; generated with SWISS-MODEL). Key residues of p37-UBX/p47-UBX for p97-interaction are labeled.

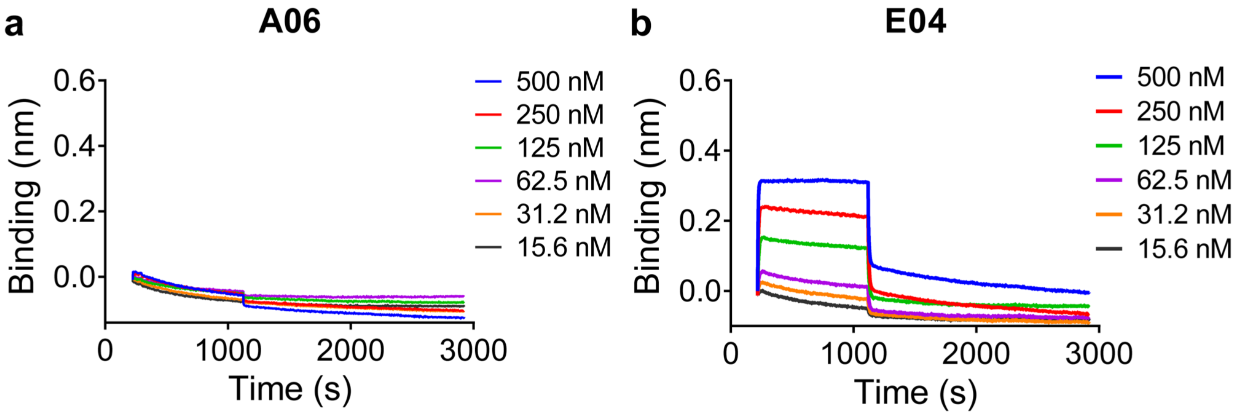

Figure S5. BLI dose response profiles of scFv-A06 and scFv-E04 to p37-UBX domain. The binding affinity of scFv-E04 for p37-UBX based on BLI results is $K_D = 3.72 \pm 0.26$ nM. Standard deviations represent $N = 2$ independent experiments. Data represent $N = 2$ independent experiments.

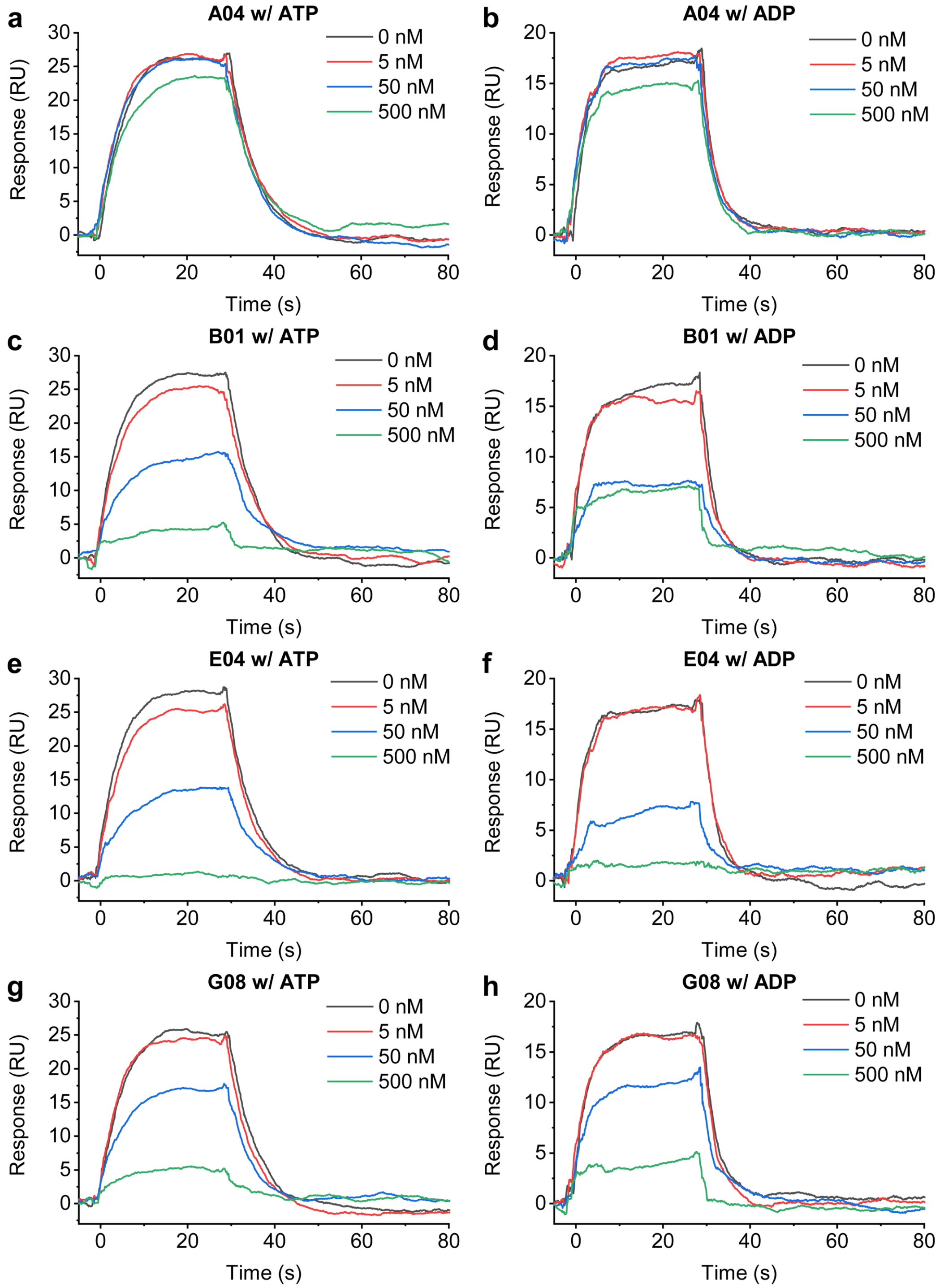

Figure S6. Surface plasmon resonance (SPR) sensorgrams for the scFv-binder/p47-UBX mixture binding to full-length human p97 in the presence of either 100 μM ATP or 100 μM ADP. In the mixture, the concentration of p47-UBX was fixed at 50 nM with an increasing concentration of scFv binders. Data represent $N = 2$ independent experiments.

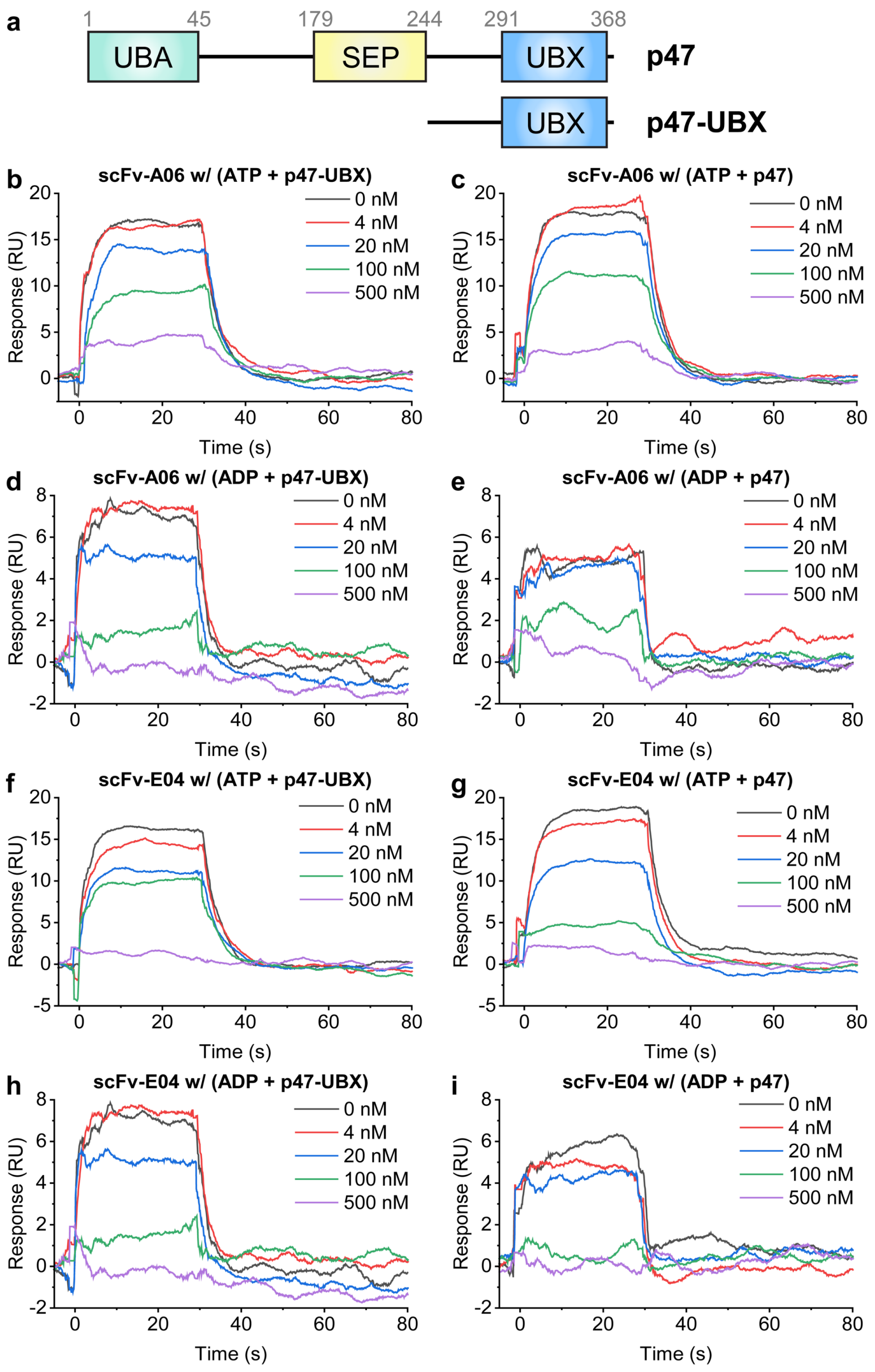

Figure S7. (a) Schematic illustration of p47 constructs in this study. (b~i) Surface plasmon resonance (SPR) sensorgrams for the scFv-binder/p47-UBX or the scFv-binder/p47 mixture binding to full-length human p97 in the presence of either 100 μM ATP or 100 μM ADP. In the mixture, the concentration of p47-UBX or p47 was fixed at 50 nM with an increasing concentration of scFv binders.

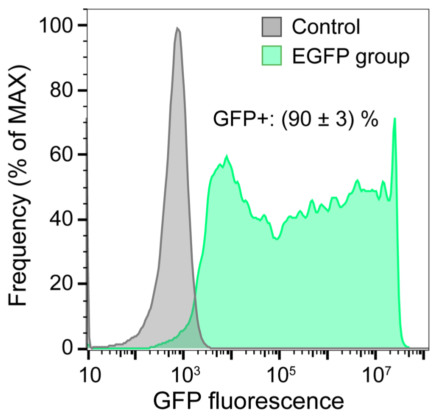

Figure S8. Flow cytometry histograms of U2OS cells transfected with EGFP-expressing plasmids using Xfect. Data represent $N = 3$ independent experiments.

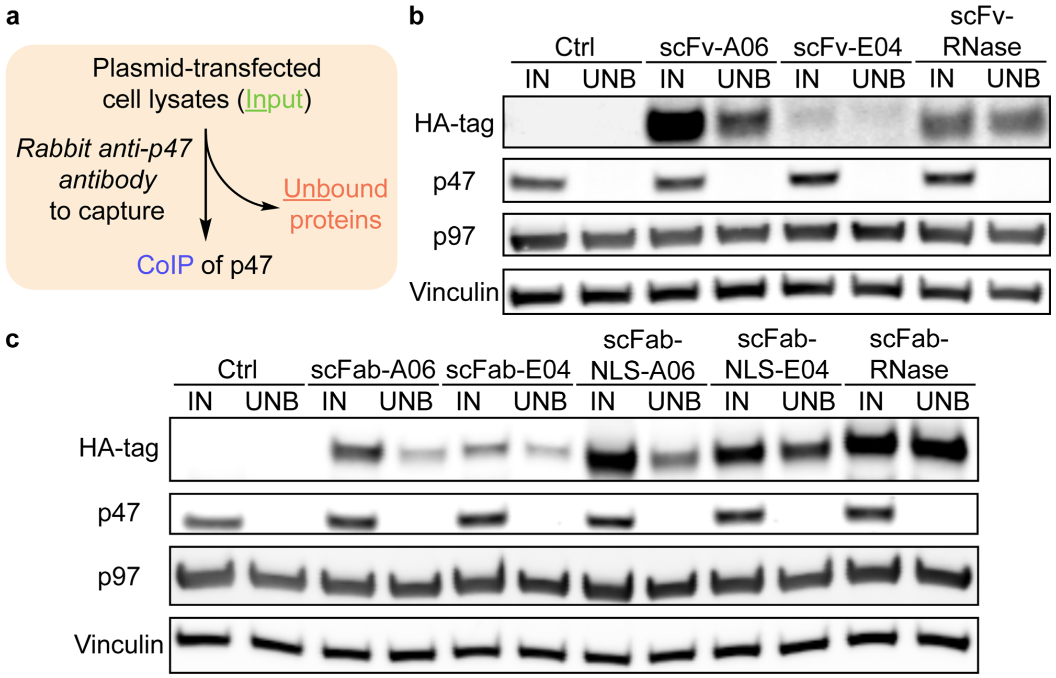

Figure S9. (a) Workflow of the co-immunoprecipitation (co-IP) and western blot analysis for cells after plasmid transfection. Western blots for HA-tag, p47, p97, and vinculin in U2OS cells after 24-h transfection of plasmids that encode (b) scFvs and (c) scFabs. For each group, input proteins in the lysates (IN) before the co-IP of p47 were compared with the unbound proteins in the lysates (UNB). Ctrl, cell-only control group without Xfect-plasmid complexes added. Data represent $N = 2$ independent experiments.

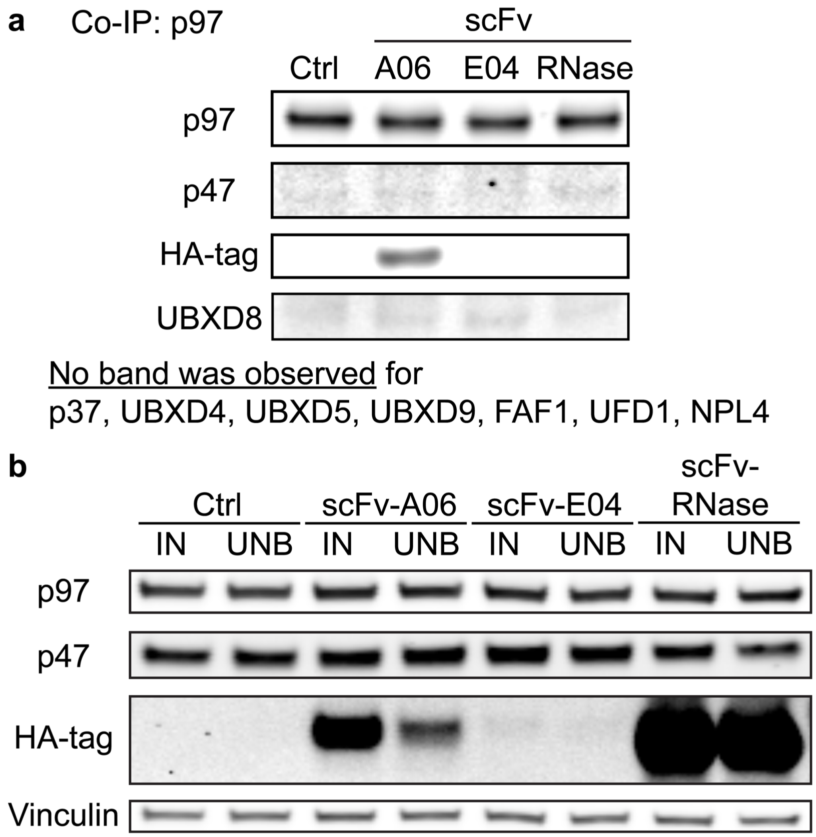

Figure S10. (a) Co-IP of p97 from U2OS cells and (b) western blots for p97, p47, HA-tag, and vinculin in U2OS cells after 24-h transfection of plasmids that encode scFvs. The interactions between p97 and its more than 30 adaptor proteins are exceptionally dynamic,[SR2] resulting in unsuccessful capture of most p97/adaptor complexes in the co-IP experiments of p97. Moreover, the formation of a trimeric complex was observed among p97, p47, and scFv-A06 upon the introduction of antibody fragment inhibitors in U2OS cells. For each group of western blots, input proteins in the lysates (IN) before the co-IP of p97 were compared with the unbound proteins in the lysates (UNB). Ctrl, cell-only control group without Xfect-plasmid complexes added.

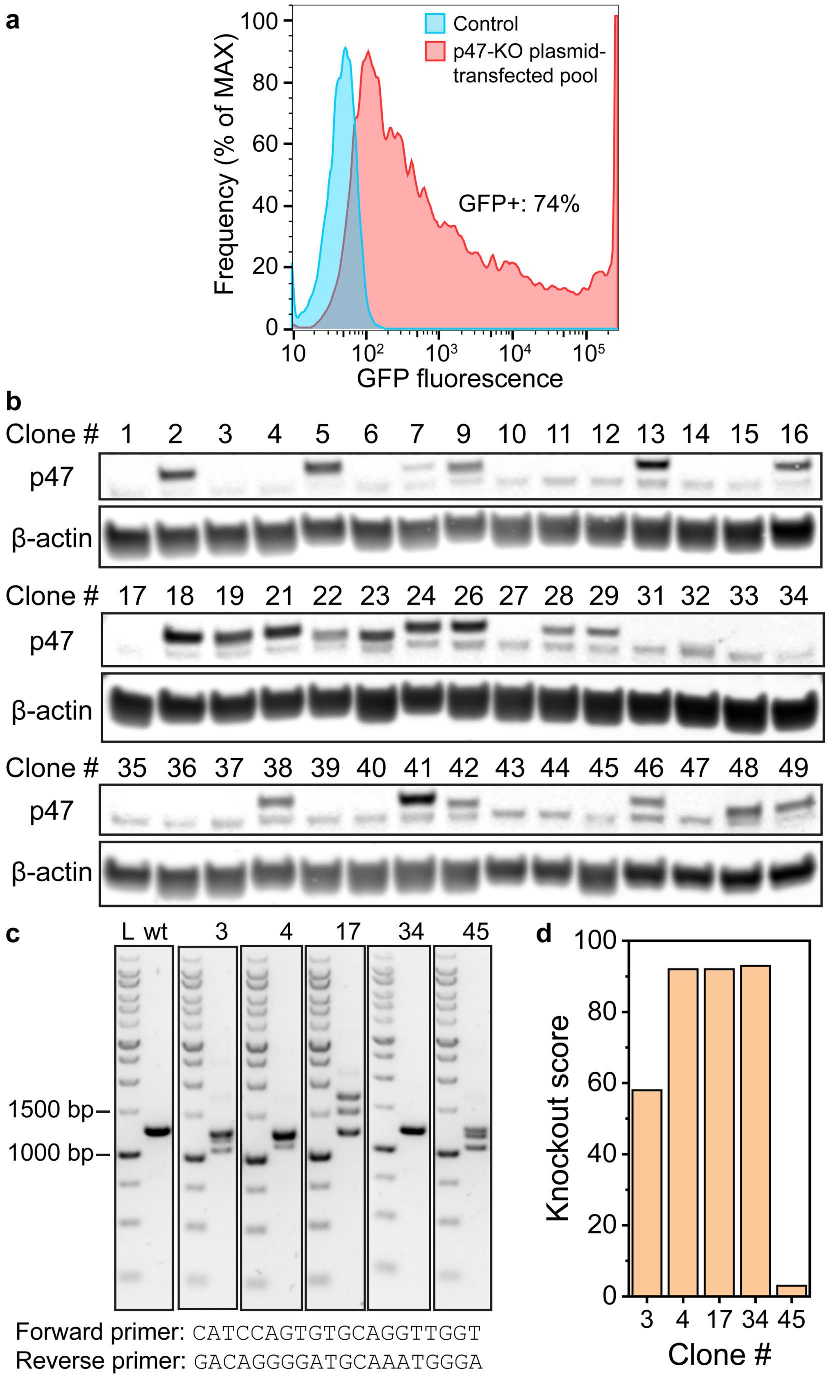

Figure S11. Generation of p47-knockout (p47-KO) U2OS cells. (a) Flow cytometry histograms of U2OS cells transfected with p47-KO plasmids (containing EGFP-encoding sequence). (b) Western blots for p47 and *β*-actin in U2OS single cell clones. (c) PCR amplification of the genomic DNA in wild-type (wt) U2OS cells and selected p47-KO U2OS clones. (d) Knockout score of selected p47-KO U2OS clones from Synthego ICE analysis on Sanger sequencing results.

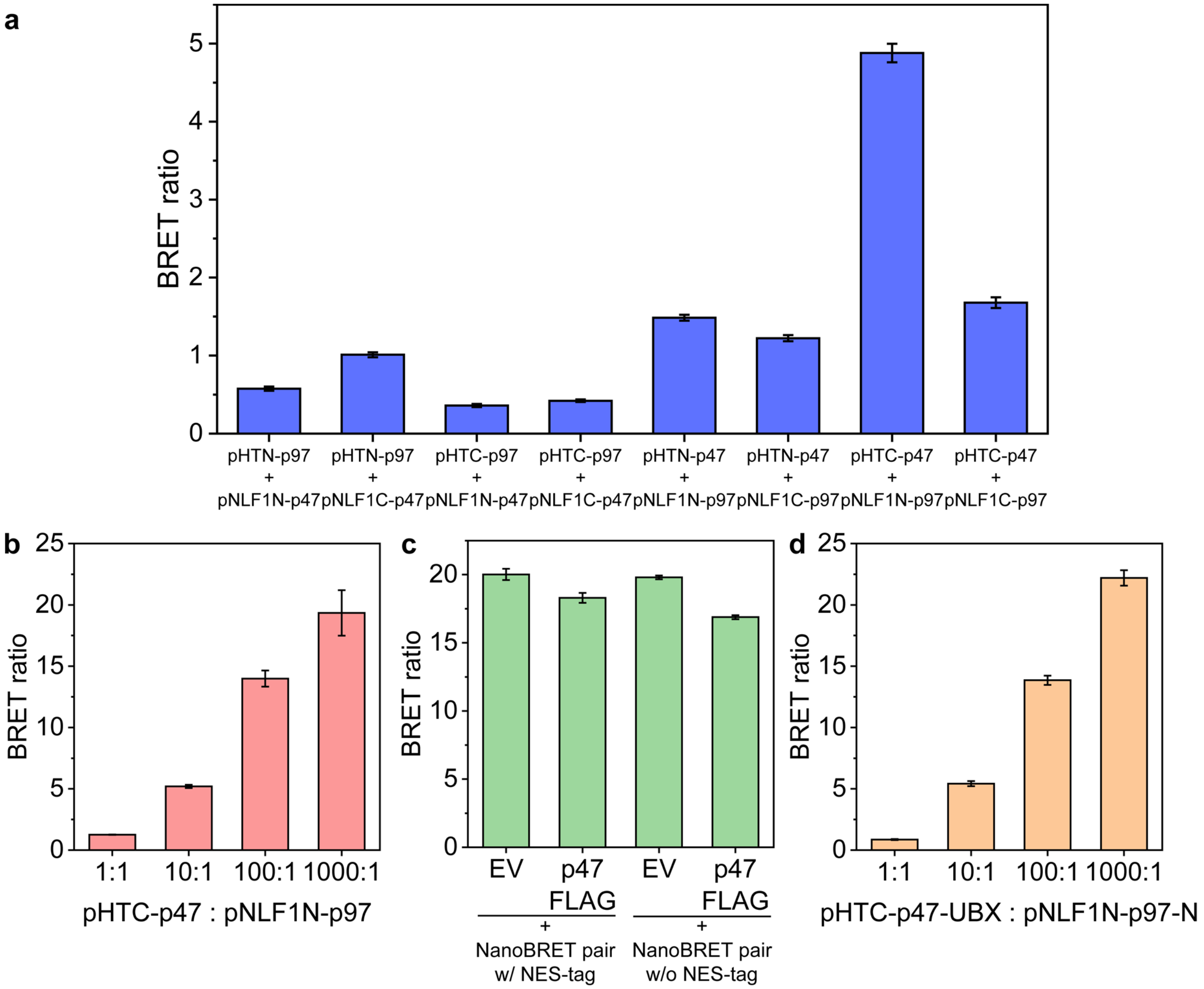

Figure S12. (a) NanoBRET assay results in wild-type U2OS cells testing all possible PPI combinations when p97 or p47 have been tagged with NanoLuc (NLF1) donor or HaloTag (HT) acceptor at either N- or C-terminus. All the combinations were transfected in a ratio of $w_{\text{plasmid}} : w_{\text{plasmid}}$ = 1:1. (b) NanoBRET assay results in wild-type U2OS cells testing varied ratios between pHTC-p47 and pNLF1N-p97. Note that all the p97 and p47 constructs in (a) and (b) were tagged with a nuclear export signal (NES). (c) NanoBRET assay results in p47-KO U2OS cells demonstrate that overexpressed p47-FLAG suppresses the BRET ratio when compared to empty vectors (EV), indicating the competition between p47-FLAG and pHTC-p47 for pNLF1N-p97 binding. (d) NanoBRET assay results in p47-KO U2OS cells testing varied ratios between pHTC-p47-UBX and pNLF1N-p97-N. N = 4 for all NanoBRET assays.

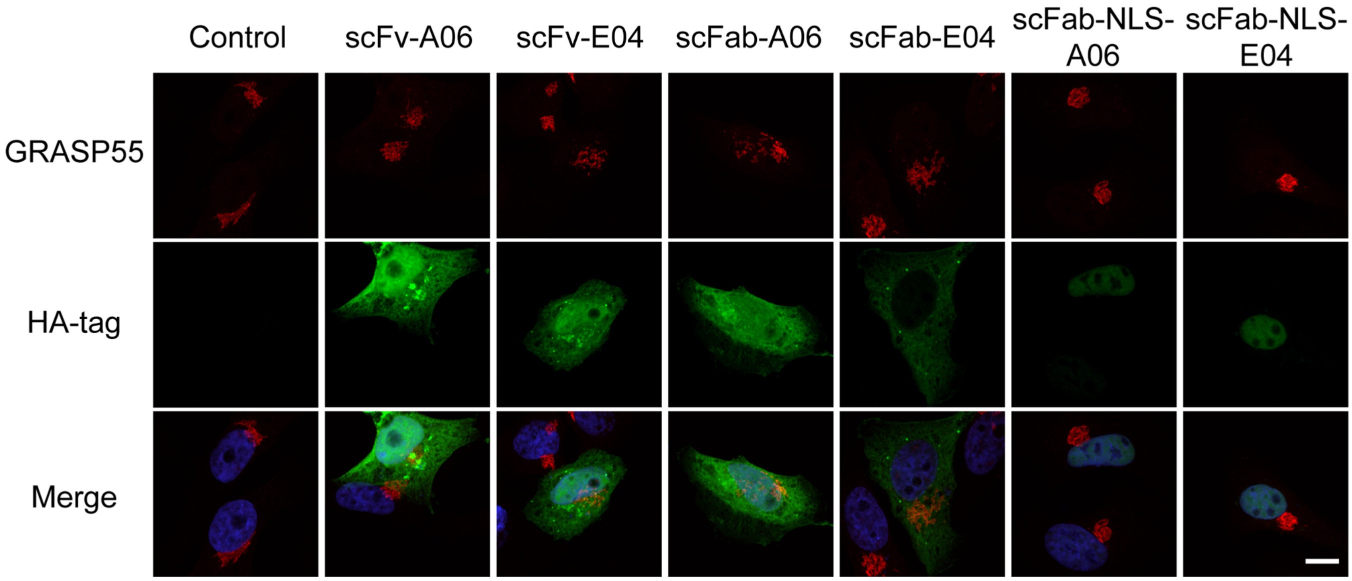

Figure S13. Representative immunofluorescence images of a Golgi marker GRASP55 in HeLa cells transfected with anti-p47-UBX antibody fragments (HA-tagged). Scale bar, 10 µm.

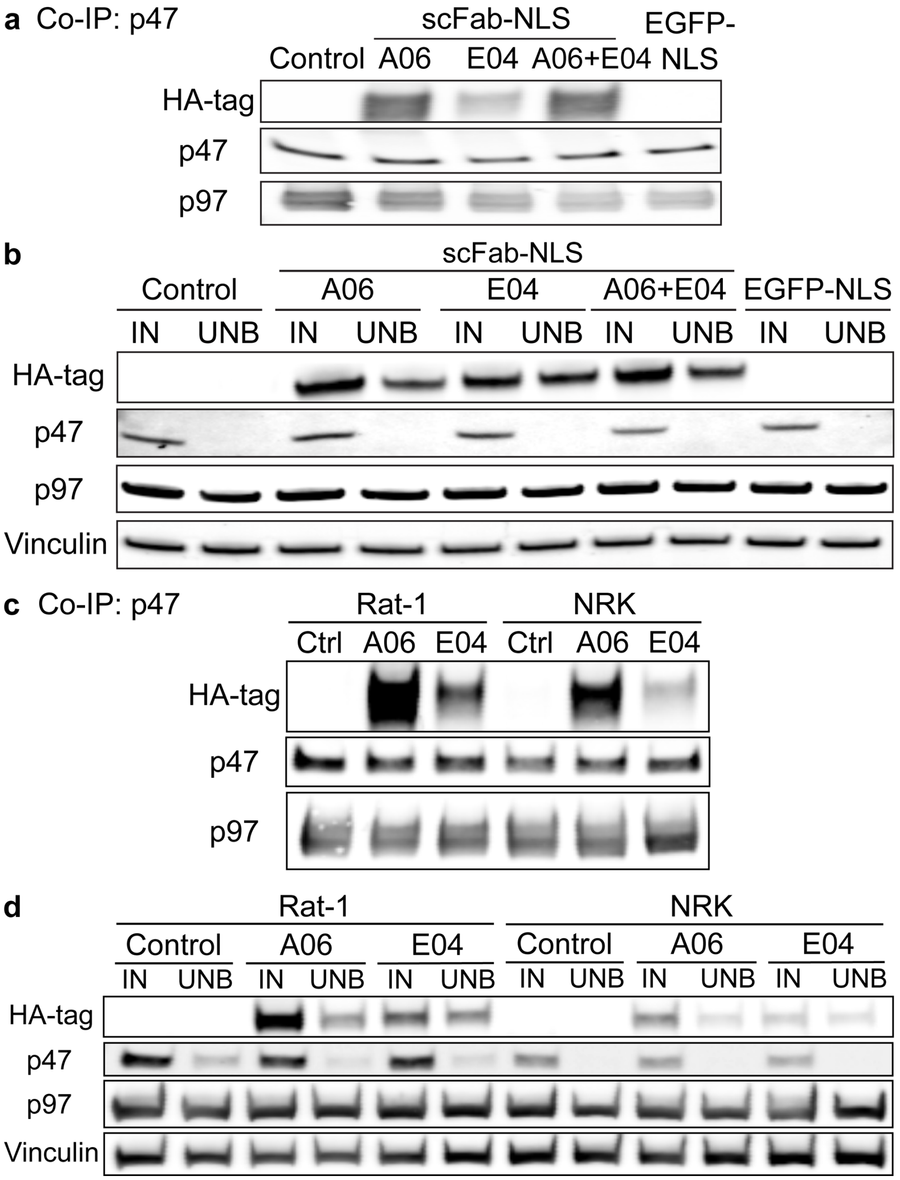

Figure S14. (a) Co-IP of p47 from HeLa cells and (b) western blots for HA-tag, p47, p97, and vinculin in HeLa cells after 24-h transfection of plasmids that encode scFab-NLS-A06, scFab-NLS-E04, scFab-NLS-A06/E04 (w_{plasmid} / w_{plasmid} = 1:1), and EGFP-NLS. (c) Co-IP of p47 from Rat-1 or NRK cells and (d) western blots for HA-tag, p47, p97, and vinculin in Rat-1 or NRK cells after 24-h transfection of plasmids that encode scFab-NLS-A06 and scFab-NLS-E04. For each group of western blots, input proteins in the lysates (IN) before the co-IP of p47 were compared with the unbound proteins in the lysates (UNB). Ctrl/Control, cell-only control group without Xfect-plasmid complexes added. Data represent N = 2 independent experiments.

2. Experimental Section

2.1. Plasmid construction

The plasmid constructs in this work were obtained by PCR amplifications of DNA fragments, followed by Gibson assemblies of the DNA fragments. P97, p47, p47-UBX, NPL4-UBXL, FAF1-UBX, and p37-UBX were cloned on *E. coli* expression vectors (pET) and expressed as biotinylated proteins as previously described.[SR3-4] These constructs for biotinylation contain a TEV (tobacco etch virus) protease cleavage site, an AviTag, and a His-tag (6 aa, HHHHHH) at either the N-terminus or the C-terminus. All the scFvs were subcloned from the Fab-phagemid into an *E. coli* expression vector pSYN1[SR5] and a mammalian expression vector pcDNA3.1. All scFabs[SR6] were designed from the scFv constructs by overlap-extension PCR[SR7] and subcloned into a pcDNA3.1 vector. The scFab-NLS constructs were subcloned from their corresponding scFab constructs with an N-terminal c-Myc NLS tag[SR8] (9 aa, PAAKRVKLD). All the constructs on pcDNA3.1 vectors in this study contain a C-terminal HA (human influenza hemagglutinin) epitope tag (9 aa, YPYDVPDYA). All constructs were sequence verified by Sanger sequencing.

2.2. Protein expression and purification

All the recombinant proteins were expressed in *E. coli* BL21(DE3) and purified as previously described.[SR3-4] Briefly, *E. coli* containing expression vectors of interest were grown in 2X YT media at 37 °C to an OD_{600} of 0.4~0.8, followed by the addition of 0.1 mM IPTG at 20 °C overnight. Bacterial cells were pelleted by centrifugation and lysed with either sonication probe or B-PER lysis buffer. Next, His-tagged proteins were purified on a Ni-NTA column, followed by size-exclusion chromatography (SEC) using a GE AKTA FPLC purification system equipped with a Superdex column. When the biotinylated tag was not needed for specific experiments, the biotinylated proteins were cleaved by TEV protease (NEB, Cat# P8112S), followed by dialysis and SEC purification. The purified fractions were identified by SDS-PAGE and characterized by mass spectrometry on a Waters Xevo QTof mass spectrometer. Fractions containing the protein of interest were concentrated by Amicon MWCO filters, flash frozen, and stored at -80 °C.

2.3. Fab-phage selection

Phage display was carried out based on previously established protocols.[SR9] Briefly, the antibody phage Library E or X7X8 were used for selection against biotinylated p47-UBX domain (antigen). After incubating the phage library with the antigen, streptavidin-functionalized magnetic beads were utilized to capture the bound phage, followed by the elution step using TEV protease. Four rounds of selections were conducted with a decreasing concentration of the antigen (500 nM, 250 nM, 100 nM, and 10 nM). Note that during the third and fourth rounds, the overnight phage culture was pre-enriched using Protein-L magnetic beads (ThermoFisher, Cat# 88849) before the antigen capture. The clones from the fourth round of selection were collected for phage ELISA analysis.

2.4. Phage ELISA

Phage ELISA was performed based on previous reports.[SR9] For each phage clone, three different conditions were tested – Direct: biotinylated p47-UBX; Competition: biotinylated p47-UBX with an equal concentration of p47-UBX (w/o biotinylation) in solution; Control: BSA (bovine serum albumin). A 384-well Nunc Maxisorp flat-bottom clear plate (Thermo Scientific, Cat# 464718) was coated with 10 $\mu g \cdot mL^{-1}$ of NeutrAvidin (Thermo Scientific, Cat# 31000) in PBS overnight at 4 °C and subsequently blocked with PBSTB (PBS buffer + 0.02% Tween-20 + 0.2% BSA) for 1 hour at room temperature. Plates were washed three times with PBS containing 0.05% Tween-20 and were washed similarly between each step. Next, 20 nM of biotinylated p47-UBX diluted in PBSTB was captured on the NeutrAvidin-coated wells (for Direct and Competition wells; PBSTB for the Control wells) for 30 min, then blocked with PBSTB + 20 μM biotin for 10 min. After washing the plate, phage supernatant diluted (5 times) in PBSTB was added for 1 hour at 4 °C for the Direct and Control groups. For the Competition groups, the phage supernatant was diluted into PBSTB with 20 nM soluble p47-UBX and incubated for 1 hour at 4 °C. Bound phage was detected by incubating with anti-M13-horseradish peroxidase conjugate (Sino Biologics, Cat#11973-MM05-H, 1:5000) for 30 min, followed by the addition of TMB substrate (VWR, Cat# 95059-156) and incubation at room temperature until signal appeared. The reaction was quenched by the addition of 1 M

phosphoric acid. The absorbance at 450 nm was measured using a SpectraMax plate reader. The ELISA data were analyzed to refine hits with the two following features: (1) Specificity = $OD_{450\,nm}$ (Direct)/$OD_{450\,nm}$ (Control) > 4; (2) Competition ratio = $OD_{450\,nm}$ (Competition)/$OD_{450\,nm}$ (Direct) < 0.5.

2.5. Biophysical characterizations

2.5.1. Bio-layer interferometry experiments (BLI)

BLI measurements were conducted with a ForteBio Octet RED384 system. Biotinylated p97 adaptor proteins (p47-UBX, NPL4-UBXL, FAF1-UBX, or p37-UBX) were immobilized to SA (streptavidin) biosensor tips (ForteBio). Varied concentrations of scFvs in PBSTB were used as analytes in 96-well microplates (Greiner Bio-One, Cat# 655209). Data were analyzed using the ForteBio Octet RED384 Data Analysis HT software and binding affinities (K_D) were determined using a 1:1 monovalent binding model.

2.5.2. Surface plasmon resonance (SPR)

The SPR experiments were carried out on a GE Biacore 4000 instrument to measure the competition of antibodies against p97/p47 interaction. Briefly, NeutrAvidin-coated sensor chips were prepared on CM5 chips as previously described.[SR4] Biotinylated p97 proteins were immobilized to 500-900 RUs (response units) by injecting 4~6 $\mu M \cdot mL^{-1}$ protein for 2 min using the immobilization buffer (10 mM HEPES, pH 7.5, 150 mM NaCl, 0.5 mM TCEP, and 0.05% Tween-20). Binding of the p47-UBX (or p47) and antibody mixture was measured at 20 °C in duplicates. In the p47-UBX (or p47) and antibody mixture, p47-UBX (or p47) was fixed at 50 nM with varied scFv concentrations (5 nM, 50 nM, and 500 nM; or 4 nM, 20 nM, 100 nM, and 500 nM). To measure binding in the presence of nucleotides, 100 μM ADP or ATP was added to the assay buffer (25 mM Tris, pH 7.5, 150 mM NaCl, 10 mM $MgCl_2$, 0.5 mM TCEP, 0.05% Tween-20, 10 μM bovine gamma globulin). Sensorgrams were processed and data were fit to a 2-site equilibrium model in Scrubber2 as described.[SR3-4]

2.6. Cell culture

All mammalian cells used in the study were cultured in a humidified atmosphere (5% CO_2) at 37 °C. U2OS cell line was purchased from ATCC (Cat# HTB-96). HeLa cell line was purchased from ATCC (Cat# CCL-2). Rat-1 and NRK cell lines were acquired from the UCSF Cell and Genome Engineering Core. Unless otherwise mentioned, cells were grown and passaged in Dulbecco's modified eagle medium + GlutaMAX (DMEM, ThermoFisher, Cat# 10567014) supplemented with 10% fetal bovine serum (FBS) and 1X antibiotics (100 U·mL^{-1} penicillin and 100 μg·mL^{-1} streptomycin). Cells were passaged until reaching ~70% confluency, with the media replaced every 2~3 days.

2.7. Immunofluorescence (IF)

A total of 20 k U2OS cells in 400 μL complete DMEM media (w/o antibiotics) were cultured in an 8-well chambered cover glass (Cellvis, Cat# C8-1.5H-N) for 24 hours prior to the experiment. The complexes between 0.12 μL Xfect transfection reagent (Takara Bio, Cat# 631318) and 0.4 μg plasmid (for the expression of scFv-A06, scFab-A06, or scFab-NLS-A06) were mixed in 6 μL Xfect reaction buffer for 10 min. After replacing the media with 100 μL fresh complete DMEM media (w/o antibiotics), the Xfect-plasmid mixture was spiked into the well and incubated at 37 °C. After 24 hours, transfected cells were washed once with cold PBS and fixed/permeated with the BD Fixation/Permeablization Kit (BD Biosciences, Cat# 554714). The cells were blocked with PBST (PBS + 0.1% Tween-20) that contains 1% BSA and 0.3 M glycine for 30 min, followed by overnight incubation at 4 °C with diluted primary antibodies [Mouse anti-HA-tag (C-terminus of antibody fragments) antibody: Cell Signaling Technology, Cat# 2367, 1:100 diluted; Rabbit anti-p47 antibody: ThermoFisher, Cat# PA5-61429, 1:200 diluted]. After washing the cells with PBS once for 5 min, the cells were incubated with diluted secondary antibodies (Goat anti-mouse antibody, Alexa Fluor 488: Abcam, Cat# ab150113, 1:1000 diluted; Goat anti-rabbit antibody, Alexa Fluor 647: Abcam, Cat# ab150083, 1:1000 diluted) for 1 h at room temperature in the dark. Note that all the primary and secondary antibodies for IF experiments were diluted in PSBT that contains 1% BSA. After washing with PBS, the cells were imaged in PBS on a Nikon Ti confocal microscope equipped with a Yokagawa CSU22

spinning disk unit. The intracellular distribution was measured with excitation wavelengths of 488 nm (Alexa Fluor 488) and 561 nm (Alexa Fluor 647).

2.8. Co-immunoprecipitation (co-IP) and western blot

Transfection and lysate preparation. Mammalian cells (a total of 120 k for U2OS and HeLa cells, 150 k for Rat-1 and NRK cells) in complete DMEM media (w/o antibiotics) were cultured in a 6-well plate (Corning, Cat# 3516) for 24 hours prior to the experiment. The complexes between 1.5 μL Xfect transfection reagent (Takara Bio, Cat# 631318) and 5 μg plasmids (for the expression of antibody fragments) were mixed in 100 μL Xfect reaction buffer for 10 min. After replacing the media with 900 μL fresh complete DMEM media (w/o antibiotics), the Xfect-plasmid mixture was added dropwise into the well and incubated at 37 °C for 24 hours. Next, the complex-containing media was aspirated and the cells were washed once with cold PBS. After aspirating the PBS, 300 μL of NP-40 lysis buffer [50 mM Tris·HCl pH 7.5, 150 mM NaCl, 0.5% NP-40 substitute (Sigma-Aldrich, Cat# 74385), 1 tablet of protease inhibitor (Sigma-Aldrich, Cat# 05892791001) per 10-mL buffer] was added to the cells and the plate was placed on an orbital shaker at 4 °C for 5 min. Each well was then thoroughly scraped with a 1-mL pipette tip. The lysate suspension in each well was collected and centrifuged at 14,000 rpm for 15 min at 4 °C. The supernatant was collected as the cell lysates for co-IP and western blot analysis.

Co-IP and western blot. The cell lysates were mixed with 3 μL rabbit anti-p47 antibodies (ThermoFisher, Cat# PA5-61429) and incubated overnight at 4 °C. Meanwhile, 50 μL Protein-A magnetic beads (ThermoFisher, Cat# 88845) were pre-washed with NP-40 lysis buffer. Next, 200 μL of the lysates were incubated with the pre-washed beads at room temperature on an orbitron rotator, and the remaining lysates were labeled as the INPUT group. After an hour of incubation, the beads were collected by a magnet (ThermoFisher, Cat# 12321D) and the lysates containing the unbound proteins were collected (the UNBOUND group). The beads with p47-bound complexes were heated at 90 °C for 5 min in 1X LDS sample buffer (ThermoFisher, Cat# B0007; diluted in NP-40 cell lysis buffer), resulting in the elution as the co-IP portion of the corresponding group. For the co-IP of p97, mouse anti-p97 antibodies (SCBT, Cat# sc-57492) and Protein A/G magnetic

beads (ThermoFisher, Cat# 88802) were used to capture the p97-containing complexes. During the elution step, the beads containing p47-bound complexes were mixed at room temperature for 10 min in 1X LDS sample buffer.

For western blots, protein levels of the INPUT and UNBOUND groups were quantified using Pierce Rapid Gold BCA Protein Assay Kit (ThermoFisher, Cat# A53225) and lysates were diluted to approximately equal concentrations with NP-40 lysis buffer, followed by heating in 1X LDS sample buffer (ThermoFisher, Cat# B0007) at 90 °C for 5 min. Equal amounts of INPUT and UNBOUND samples (typically 8 μg total protein per lane) were loaded into lanes of a 12-, 15-, or 17-well Bolt 4~12% bis-tris gels (ThermoFisher, Cat# NW04122BOX, NW04125BOX, or NW04127BOX) and run at 90 V constant for 100 min. For co-IP samples, ~15 μL elution from each group was loaded in each lane. Next, protein was transferred to a PVDF membrane within the iBlot 2 mini transfer stacks (ThermoFisher, Cat # IB24002) using the iBlot 2 gel transfer device (ThermoFisher, Cat# IB21001; Condition: 20 V, 7 min). The transferred PVDF membrane was blocked with TBS blocking buffer (LI-COR Biosciences, Cat# 927-60001) for an hour at room temperature. The membrane was probed with primary antibodies that were diluted in TBS blocking buffer (contains 0.1% Tween-20) overnight at 4°C. After washing with 1X TBST (50 mM Tris·HCl, 150 mM NaCl, 0.1% Tween-20), the membrane was incubated in secondary antibodies that were diluted in TBS blocking buffer (contains 0.1% Tween-20) for 1 h at room temperature. After washing with 1X TBST, the membranes were imaged on an Odyssey CLx infrared imaging system (LI-COR Biosciences).

List of primary and secondary antibodies for co-IP and western blot.

Mouse anti-HA-tag antibody, CST, Cat# 2367S, 1:1000 diluted;

Rabbit anti-HA-tag antibody, CST, Cat# 3724S, 1:1000 diluted;

Mouse anti-p47 antibody, SCBT, Cat# sc-376614, 1:500 diluted;

Rabbit anti-p47 antibody, ThermoFisher, Cat# PA5-61429, 1:1000 diluted;

Mouse anti-p97 antibody, SCBT, Cat# sc-57492, 1:1000 diluted;

Rabbit anti-p97 antibody, Proteintech, Cat# 10736-1-AP, 1:1000 diluted;

Rabbit anti-UBXD8 antibody, CST, Cat# 34945, 1:1000 diluted;

Rabbit anti-p37 antibody, ThermoFisher, Cat# PA5-60969, 1:1000 diluted;

Rabbit anti-UBXD4 antibody, Origene, Cat# TA350624, 1:1000 diluted;

Rabbit anti-UBXD5 antibody, Proteintech, Cat# 13109-1-AP, 1:1000 diluted;

Rabbit anti-UBXD9 antibody, CST, Cat# 2049, 1:1000 diluted;

Rabbit anti-FAF1 antibody, CST, Cat# 4932, 1:1000 diluted;

Rabbit anti-UFD1 antibody, CST, Cat# 13789, 1:1000 diluted;

Rabbit anti-NPL4 antibody, CST, Cat# 13489, 1:1000 diluted;

Rabbit anti-vinculin antibody, CST, Cat# 13901S, 1:2000 diluted;

Mouse anti-β-actin antibody, CST, Cat# 3700S, 1:1000 diluted.

Goat-anti-rabbit secondary antibody, AzureSpectra 700 conjugates, Azure Biosystems, Cat# AC2128, 1:5000 diluted;

Goat-anti-mouse secondary antibody, AzureSpectra 700 conjugates, Azure Biosystems, Cat# AC2129, 1:5000 diluted;

Goat-anti-rabbit secondary antibody, AzureSpectra 800 conjugates, Azure Biosystems, Cat# AC2134, 1:5000 diluted;

Goat-anti-mouse secondary antibody, AzureSpectra 800 conjugates, Azure Biosystems, Cat# AC2135, 1:5000 diluted.

2.9. NanoBRET p97/p47 PPI assay

2.9.1. Generation of p47-knockout (p47-KO) U2OS cells

Plasmid transfection. A total of 65 k U2OS cells in complete DMEM media (w/o antibiotics) were cultured in a 12-well plate (Corning, Cat# 3513) for 24 hours prior to the experiment. The complexes between 0.75 μL Xfect transfection reagent (Takara Bio, Cat# 631318) and 2.5 μg p47-KO plasmid (Santa Cruz Biotechnology, Cat# sc-402328) were mixed in 50 μL Xfect reaction buffer for 10 min. After replacing the media with 450 μL fresh complete DMEM media (w/o antibiotics), the

Xfect-plasmid mixture was added dropwise into the well and incubated at 37 °C. After 24 hours, the complex-containing media was replaced with 1-mL fresh complete DMEM media. Transfected cells were allowed to grow for another 2 days.

Cell sorting and expansion. Next, trypsinized cells were suspended in sorting buffer (PBS containing 5% FBS). A selected population of GFP-positive cells were sorted as single clones into 96-well plates (Corning, Cat# 3628) using a BD FACS Aria II sorter. The sorted single-cell clones were cultured in complete DMEM/F12 media (ThermoFisher, Cat# 10565018, supplemented with 10% FBS) for 2 weeks, with fresh DMEM/F12 media replaced on Day 5 and every 3 days afterwards. After 2 weeks, successfully expanded clones were detached from 96-well plates and further expanded in 6-well plates until at least 80% confluency was reached.

Identification and characterization of p47-KO clones. Around 50 clones were successfully expanded to the 6-well plate stage. These clones were subsequently harvested and analyzed by western blots. Five clones (C3, C4, C17, C34, and C45) without p47 expression were selected for genomic analysis. The genomic DNA of these clones were collected by a Quick-DNA miniprep plus kit (Zymo Research, Cat# D4069) and PCR amplified with Q5 polymerase (Figure S11). Note that the primers for the PCR reaction were designed 500~600 bp upstream and downstream of the targeting site of p47-KO plasmid (Santa Cruz Biotechnology, Cat# sc-402328). The PCR product of these five clones along with wild-type U2OS was cleaned up (QIAGEN, Cat# 28104) and assessed by Sanger sequencing, followed by the Synthego ICE Analysis (https://ice.synthego.com/) to compare the knockout score. Clone 34 was labeled as the p47-KO U2OS cells and used for the NanoBRET assays.

2.9.2. Plasmid design for the NanoBRET assay

All the plasmids for the p97/p47 NanoBRET assays were constructed based on the vectors provided by the NanoBRET PPI starter system (Promega, Cat# N1811). The initial test was to screen all the possible combinations of p97/p47 pairs and find the pair that demonstrates the highest BRET signal. In this step, both p97 and p47 were tagged with a nuclear export signal (NES)[SR10] at the C-terminus. The NanoLuc (NLF1) donor and HaloTag (HT) acceptor were tagged on either the N-terminus or the C-terminus of p97-NES or p47-NES, forming eight different constructs: pHTN-p97,

pHTC-p97, pNLF1N-p97, pNLF1C-p97, pHTN-p47, pHTC-p47, pNLF1N-p47, and pNLF1C-p47. Eight different combinations from the above eight constructs were tested for NanoBRET assays. Among the combinations ($w_{\text{plasmid}}/w_{\text{plasmid}}$ = 1:1), the pair of pNLF1N-p97 (donor) and pHTC-p47 (acceptor) generated the highest BRET ratio in wild-type U2OS cells. Next, varied ratios of plasmids for pHTC-p47/pNLF1N-p97 were tested (1:1, 10:1, 100:1, and 1000:1) for NanoBRET assays, and 100:1 was selected as the ratio for the assay because of high BRET ratio and low standard deviation. Another two pairs of p97/p47 constructs were also generated: (1) Full-length p97 and p47 without NES tag; (2) p97-N domain with NLF1 tagged on the N-terminus (pNLF1N-p97-N) and p47-UBX domain with HT tagged on the C-terminus (pHTC-p47-UBX). These two pairs generated similar BRET signals when compared to the full-length constructs with the NES tag (Figure S12).

2.9.3. General procedures of the NanoBRET assay

A total of 120 k U2OS cells (wild-type or p47-KO) in complete DMEM media (w/o antibiotics) were cultured in a 6-well plate (Corning, Cat# 3516) for 24 hours prior to the experiment. The complexes between 1.5 μL Xfect transfection reagent (Takara Bio, Cat# 631318) and 5 μg plasmids [5 μg NanoBRET pair plasmids in total, or 2.5 μg NanoBRET pair plasmids along with 2.5 μg plasmids for antibodies/empty vector (EV; ThermoFisher, Cat# V79020)/p47-FLAG] were mixed in 100 μL Xfect reaction buffer for 10 min. After replacing the media with 900 μL fresh complete DMEM media (w/o antibiotics), the Xfect-plasmid mixture was added dropwise into the well and incubated at 37 °C. After 24 hours, each group of the transfected cells were detached and resuspended in FluoroBrite DMEM (ThermoFisher, Cat# A1896701; supplemented with 10% FBS, 1X antibiotics, and 1X GlutaMAX). Next, ~24 k transfected cells in 100 μL FluoroBrite DMEM were seeded in 96-well plates (Corning, Cat# 3610). In each well, the experimental samples were added with 0.1 μL stock solution of the HaloTag NanoBRET 618 Ligand (the "+ligand" group), and the no-acceptor controls were added with 0.1 μL DMSO (the "-ligand" group). After incubating at 37 °C for 6 hours, 25 μL 5X solution NanoBRET Nano-Glo Substrate reagent in FluoroBrite DMEM was spiked into each well and immediately measured the luminescence on a

SpectraMax iD5 plate reader (LUM; Endpoint; User filter, Lm1: 447 nm, Lm2: 610 nm; Integration time: 1000 ms; Read height: 0.67 mm). The BRET ratio was calculated according to the manual of the NanoBRET PPI starter system.

2.10. Quantitative Golgi assays

2.10.1. IF for Golgi fragmentation

HeLa cells were seeded onto the poly-lysine-coated coverslips (ThermoFisher, Cat# 12-545-81). Cells at 50~60% confluency were transfected with plasmids that express anti-p47-UBX antibody fragments using Lipofectamine 2000 Reagent (Invitrogen, Cat# 11668019). After 24 h, cells were fixed with 4% paraformaldehyde (ThermoFisher, Cat# AC416780010) for 15 min, quenched with 50 mM NH_4Cl (ThermoFisher, Cat# A661-500) in PBS for 10 min, and permeabilized with 0.2% Triton X-100 (ThermoFisher, Cat# AC215682500) for 10 min. Next, cells were blocked with gelatin blocking buffer in PBS for 30 min at room temperature, incubated with primary antibodies (Rabbit anti-GRASP55 antibody, ProteinTech Group, Cat# 10598-1-AP, 1:200 diluted; Mouse anti-HA antibody, Sigma-Aldrich, Cat# H9658, 1:200 diluted) at 4 °C overnight and secondary antibodies [Fluorescein (FITC)-AffiniPure goat anti-mouse IgG (H+L), Jackson ImmunoResearch, Cat# 115-095-003, 1:100 diluted; Rhodamine (TRITC) AffiniPure goat anti-rabbit IgG (H+L), Jackson ImmunoResearch, Cat# 111-025-003, 1:500 diluted] at room temperature for 1 h. Hoechst 33258 (ThermoFisher, Cat# 5117) was used to stain the nuclear DNA. Images were taken with a 60X oil objective on a Nikon ECLIPSE Ti2 Confocal microscope and processed with maximum projection (Figure 4a, S13). Quantifications were performed to calculate Golgi items and area of selected ROIs using the Nikon analysis software. Cells with 15 or more Golgi items were regarded as the ones with fragmented Golgi (Figure 4b~d).

2.10.2. Golgi reassembly assay

The Golgi reassembly assay was performed as previously described.[SR11-12] Briefly, 100 μg rat liver Golgi membranes were treated with 5 mg mitotic cytosol in the presence of ATP regeneration system at 37 °C for 20 min. Mitotic Golgi fragments (MGFs) were isolated to remove the mitotic cytosol by centrifugation in 0.4 M sucrose (American Bioanalytical,

Cat# AB01900-01000) using a TLA55 rotor at 55,000 rpm for 20 min. For the reassembly process, 20 μg MGFs were incubated with 400 μg interphase cytosol alone or interphase cytosol with 3.1 μM recombinant scFv-A04 or scFv-06 for 1 h at 37 °C. Mitotic and interphase cytosols were prepared from HeLa S3 cells (ATCC, Cat# CCL-2.2). Golgi membranes were fixed in 2% glutaraldehyde solution (Electron Microscopy Sciences, Cat# 16220), washed three times with 0.1 M sodium cacodylate, and post-fixed on ice in 1% (wt./vol.) reduced osmium tetroxide, 0.1 M sodium cacodylate and 1.5% (wt./vol.) cyanoferrate. Golgi membranes were rinsed and processed for successive dehydration and embedding as previously described.[SR12] Resin blocks were cut to ultrathin (50~70 nm) sections with a diamond knife and mounted on Formvar-coated copper grids. Grids were double contrasted with 2% uranyl acetate for 2 min and then with lead citrate for 2 min, followed by an excessive wash with water. Images were captured at 8,000X magnification on a JEOL JEM-1400 transmission electron microscope (Figure 4).

3. Supplementary sequences

Full-length p97-C-AviTag: Full-length human p97 with AviTag on the C-terminus.[SR4]

Full-length human p47: Full-length human p47 with HisTag and AviTag on the N-terminus.[SR4]

pBirAcm: An IPTG inducible plasmid containing the BirA gene engineered into pACYC184; Avidity, Cat# AVB99.

p47-UBX: p47 (244-370)—TEV cleavage sequence—AviTag—HisTag (Vector backbone: pET)

MVKPKGAFKAFTGEGQKLGSTAPQVLSTSSPAQQAENEAKASSSILIDESEPTTNIQIRLADGGRLVQK
FNHSHRISDIRLFIVDARPAMAATSFILMTTFPNKELADESQTLKEANLLNAVIVQRLTTSENLYFQGG
GLNDIFEAQKIEWHEASHHHHHH

NPL4-UBXL: NPL4 (1-96)—TEV cleavage sequence—AviTag—HisTag (Vector backbone: pET)

MAESIIIRVQSPDGVKRITATKRETAATFLKKVAKEFGFQNNGFSVYINRNKTGEITASSNKSLNLLKI
KHGDLLFLFPSSLAGPSSEMETSVPPGTSENLYFQGGGLNDIFEAQKIEWHEASHHHHHH

FAF1-UBX: FAF1 (571-650)—TEV cleavage sequence—AviTag—HisTag (Vector backbone: pET)

MSEPVSKLRIRTPSGEFLERRFLASNKLQIVFDFVASKGFPWDEYKLLSTFPRRDVTQLDPNKSLLEVK
LFPQETLFLEAKETSENLYFQGGGLNDIFEAQKIEWHEASHHHHHH

p37-UBX: p37 (206-331)—TEV cleavage sequence—AviTag—HisTag (Vector backbone: pET)

MIKPRLRFKAFSGEGQKLGSLTPEIVSTPSSPEEEDKSILNAVVLIDDSVPTTKIQIRLADGSRLIQRF
NSTHRILDVRNFIVQSRPEFAALDFILVTSFPNKELTDESLTLLEADILNTVLLQQLKTSENLYFQGGG
LNDIFEAQKIEWHEASHHHHHH

scFv-A04: pelB signal sequence—V_H—24aa linker—V_L—Myc-Tag—His-Tag (Vector backbone: pSYN1)

MKYLLPTAAAGLLLLAAQPAMA↑QVQLVESGGGLVQPGGSLRLSCAASGFNVYSSSIHWVRQAPGKGLE
WVASIYPSYGSTYYADSVKGRFTISADTSKNTAYLQMNSLRAEDTAVYYCARSYEWKYSYYRGGFDYWG
QGTLVTVSSASSGGSTSGSGKPGSGEGSSGSARDIVMSDIQMTQSPSSLSASVGDRVTITCRASQSVSS
AVAWYQQKPGKAPKLLIYSASSLYSGVPSRFSGSRSGTDFTLTISSLQPEDFATYYCQQGYYLPVTFGQ
GTKVEIKRAAAEQKLISEEDLNGAAHHHHHH

scFv-A06: pelB signal sequence—V_H—24aa linker—V_L—Myc-Tag—His-Tag (for bacterial expression; Vector backbone: pSYN1)

MKYLLPTAAAGLLLLAAQPAMA↑QVQLVESGGGLVQPGGSLRLSCAASGFNISSYSMHWVRQAPGKGLE
WVASISSSYGSTYYADSVKGRFTISADTSKNTAYLQMNSLRAEDTAVYYCARSDWYYGDAWGVPYYAFD
YWGQGTLVTVSSASSGGSTSGSGKPGSGEGSSGSARDIVMSDIQMTQSPSSLSASVGDRVTITCRASQS
VGSALAWYQQKPGKAPKLLIYSASSLYSGVPSRFSGSRSGTDFTLTISSLQPEDFATYYCQQSWFLITF
GQGTKVEIKRAAAEQKLISEEDLNGAAHHHHHH

scFv-B01: pelB signal sequence—V_H—24aa linker—V_L—Myc-Tag—His-Tag (Vector backbone: pSYN1)

MKYLLPTAAAGLLLLAAQPAMA↑QVQLVESGGGLVQPGGSLRLSCAASGFNISSYYMHWVRQAPGKGLE
WVASIYPSYSSTSYADSVKGRFTISADTSKNTAYLQMNSLRAEDTAVYYCARSSDYDRYWYWSYDAFDY
WGQGTLVTVSSASSGGSTSGSGKPGSGEGSSGSARDIVMSDIQMTQSPSSLSASVGDRVTITCRASQSV
GSALAWYQQKPGKAPKLLIYSASSLYSGVPSRFSGSRSGTDFTLTISSLQPEDFATYYCQQSWDLFTFG
QGTKVEIKRAAAEQKLISEEDLNGAAHHHHHH

scFv-E04: pelB signal sequence—V_H—24aa linker—V_L—Myc-Tag—His-Tag (for bacterial expression; Vector backbone: pSYN1)

MKYLLPTAAAGLLLLAAQPAMA↑QVQLVESGGGLVQPGGSLRLSCAASGFNFSSYSIHWVRQAPGKGLE
WVASISPYYGSTYYADSVKGRFTISADTSKNTAYLQMNSLRAEDTAVYYCARYKYYMSGRYTWALDYWG
QGTLVTVSSASSGGSTSGSGKPGSGEGSSGSARDIVMSDIQMTQSPSSLSASVGDRVTITCRASQSVSS
AVAWYQQKPGKAPKLLIYSASSLYSGVPSRFSGSRSGTDFTLTISSLQPEDFATYYCQQEFFVPFTFGQ
GTKVEIKRAAAEQKLISEEDLNGAAHHHHHH

scFv-G08: pelB signal sequence—V_H—24aa linker—V_L—Myc-Tag—His-Tag (Vector backbone: pSYN1)

MKYLLPTAAAGLLLLAAQPAMA↑QVQLVESGGGLVQPGGSLRLSCAASGFNISYSYIHWVRQAPGKGLE
WVAYIYPYSGSTYYADSVKGRFTISADTSKNTAYLQMNSLRAEDTAVYYCARDPHWAMDYWGQGTLVTV
SSASSGGSTSGSGKPGSGEGSSGSARDIVMSDIQMTQSPSSLSASVGDRVTITCRASQSVGSALAWYQQ
KPGKAPKLLIYSASSLYSGVPSRFSGSRSGTDFTLTISSLQPEDFATYYCQQSFSSVPFTFGQGTKVEI
KRAAAEQKLISEEDLNGAAHHHHHH

scFv-A06: V_H—24aa linker—V_L—Myc-Tag—HA-Tag (for mammalian expression; Vector backbone: pcDNA3.1)

MQVQLVESGGGLVQPGGSLRLSCAASGFNISSYSMHWVRQAPGKGLEWVASISSSYGSTYYADSVKGRF
TISADTSKNTAYLQMNSLRAEDTAVYYCARSDWYYGDAWGVPYYAFDYWGQGTLVTVSSASSGGSTSGS
GKPGSGEGSSGSARDIVMSDIQMTQSPSSLSASVGDRVTITCRASQSVGSALAWYQQKPGKAPKLLIYS

ASSLYSGVPSRFSGSRSGTDFTLTISSLQPEDFATYYCQQSWFLITFGQGTKVEIKRAAAEQKLISEED
LYPYDVPDYA

scFv-E04: V_H—24aa linker—V_L—Myc-Tag—HA-Tag (for mammalian expression; Vector backbone: pcDNA3.1)

MQVQLVESGGGLVQPGGSLRLSCAASGFNFSSYSIHWVRQAPGKGLEWVASISPYYGSTYYADSVKGRF
TISADTSKNTAYLQMNSLRAEDTAVYYCARYKYYMSGRYTWALDYWGQGTLVTVSSASSGGSTSGSGKP
GSGEGSSGSARDIVMSDIQMTQSPSSLSASVGDRVTITCRASQSVSSAVAWYQQKPGKAPKLLIYSASS
LYSGVPSRFSGSRSGTDFTLTISSLQPEDFATYYCQQEFFVPFTFGQGTKVEIKRAAAEQKLISEEDLY
PYDVPDYA

scFv-RNase A:[SR13] V_H—15aa linker—V_L—HA-Tag (Vector backbone: pcDNA3.1)

MAAQPADVQLQESGPGLVKPSQSLSLTCTVTGYSITSDYAWNWIRQFPGNKLEWMGYISHSGSTGYNPS
LKSRISITRDTSKNQFFLQLNSVTTEDTATYYCARGGKNWDAYWGQGLWSLSSGGGGSGGGGSGGGGSD
IKMTQSPSSMYAFLGERVTITCKASQDINSYLSWFQQKPGKSPKTLIYRANRLVDGVPSRFSGSGSGQD
YSLTISSLEYEDMGIYYCLQYDELPFTFGSGTKLEIKAAAINYPYDVPDYAS

scFab-A06: V_L—60aa linker—V_H—HA-Tag (Vector backbone: pcDNA3.1)

MSDIQMTQSPSSLSASVGDRVTITCRASQSVGSALAWYQQKPGKAPKLLIYSASSLYSGVPSRFSGSRS
GTDFTLTISSLQPEDFATYYCQQSWFLITFGQGTKVEIKRTVAAPSVFIFPPSDSQLKSGTASVVCLLN
NFYPREAKVQWKVDNALQSGNSQESVTEQDSKDSTYSLSSTLTLSKADYEKHKVYACEVTHQGLSSPVT
KSFNRGECGGSSGSGSGSTGTSSSGTGTSAGTTGTSASTSGSGSGGGGGSGGGGSAGGTATAGASSGSE
VQLVESGGGLVQPGGSLRLSCAASGFNISSYSMHWVRQAPGKGLEWVASISSSYGSTYYADSVKGRFTI
SADTSKNTAYLQMNSLRAEDTAVYYCARSDWYYGDAWGVPYYAFDYWGQGTLVTVSSASTKGPSVFPLA
PSSKSTSGGTAALGCLVKDYFPEPVTVSWNSGALTSGVHTFPAVLQSSGLYSLSSVVTVPSSSLGTQTY
ICNVNHKPSNTKVDKKVEPKSCDKTHTGGSGSAGGLNDIFEAQKIEWHEEIKRAAAEQKLISEEDLYPY
DVPDYA

scFab-E04: V_L—60aa linker—V_H—HA-Tag (Vector backbone: pcDNA3.1)

MSDIQMTQSPSSLSASVGDRVTITCRASQSVSSAVAWYQQKPGKAPKLLIYSASSLYSGVPSRFSGSRS
GTDFTLTISSLQPEDFATYYCQQEFFVPFTFGQGTKVEIKRTVAAPSVFIFPPSDSQLKSGTASVVCLL
NNFYPREAKVQWKVDNALQSGNSQESVTEQDSKDSTYSLSSTLTLSKADYEKHKVYACEVTHQGLSSPV
TKSFNRGECGGSSGSGSGSTGTSSSGTGTSAGTTGTSASTSGSGSGGGGGSGGGGSAGGTATAGASSGS
EVQLVESGGGLVQPGGSLRLSCAASGFNFSSYSIHWVRQAPGKGLEWVASISPYYGSTYYADSVKGRFT
ISADTSKNTAYLQMNSLRAEDTAVYYCARYKYYMSGRYTWALDYWGQGTLVTVSSASTKGPSVFPLAPS
SKSTSGGTAALGCLVKDYFPEPVTVSWNSGALTSGVHTFPAVLQSSGLYSLSSVVTVPSSSLGTQTYIC
NVNHKPSNTKVDKKVEPKSCDKTHTGGSGSAGGLNDIFEAQKIEWHEEIKRAAAEQKLISEEDLYPYDV
TDYA

scFab-RNase A: V_L—C_L—60aa linker—V_H—C_H—HA-Tag (Vector backbone: pcDNA3.1)

MDIKMTQSPSSMYAFLGERVTITCKASQDINSYLSWFQQKPGKSPKTLIYRANRLVDGVPSRFSGSGSG
QDYSLTISSLEYEDMGIYYCLQYDELPFTFGSGTKLEIKAAAEIKRTVAAPSVFIFPPSDSQLKSGTAS
VVCLLNNFYPREAKVQWKVDNALQSGNSQESVTEQDSKDSTYSLSSTLTLSKADYEKHKVYACEVTHQG
LSSPVTKSFNRGECGGSSGSGSGSTGTSSSGTGTSAGTTGTSASTSGSGSGGGGGSGGGGSAGGTATAG
ASSGSAAQPADVQLQESGPGLVKPSQSLSLTCTVTGYSITSDYAWNWIRQFPGNKLEWMGYISHSGSTG
YNPSLKSRISITRDTSKNQFFLQLNSVTTEDTATYYCARGGKNWDAYWGQGLWSLSSTVSSASTKGPSV
FPLAPSSKSTSGGTAALGCLVKDYFPEPVTVSWNSGALTSGVHTFPAVLQSSGLYSLSSVVTVPSSSLG
TQTYICNVNHKPSNTKVDKKVEPKSCDKTHTGGSGSAGGLNDIFEAQKIEWHEINYPYDVPDYAS

scFab-A06-NLS: c-Myc-NLS—V_L—C_L—60aa linker—V_H—C_H—HA-Tag (Vector backbone: pcDNA3.1)

MPAAKRVKLDLEMSDIQMTQSPSSLSASVGDRVTITCRASQSVGSALAWYQQKPGKAPKLLIYSASSLY
SGVPSRFSGSRSGTDFTLTISSLQPEDFATYYCQQSWFLITFGQGTKVEIKRTVAAPSVFIFPPSDSQL
KSGTASVVCLLNNFYPREAKVQWKVDNALQSGNSQESVTEQDSKDSTYSLSSTLTLSKADYEKHKVYAC
EVTHQGLSSPVTKSFNRGECGGSSGSGSGSTGTSSSGTGTSAGTTGTSASTSGSGSGGGGGSGGGGSAG
GTATAGASSGSEVQLVESGGGLVQPGGSLRLSCAASGFNISSYSMHWVRQAPGKGLEWVASISSSYGST
YYADSVKGRFTISADTSKNTAYLQMNSLRAEDTAVYYCARSDWYYGDAWGVPYYAFDYWGQGTLVTVSS
ASTKGPSVFPLAPSSKSTSGGTAALGCLVKDYFPEPVTVSWNSGALTSGVHTFPAVLQSSGLYSLSSVV
TVPSSSLGTQTYICNVNHKPSNTKVDKKVEPKSCDKTHTGGSGSAGGLNDIFEAQKIEWHEEIKRAAAI
NYPYDVPDYAS

scFab-E04-NLS: c-Myc-NLS—V_L—C_L—60aa linker—V_H—C_H—HA-Tag (Vector backbone: pcDNA3.1)

MPAAKRVKLDLEMSDIQMTQSPSSLSASVGDRVTITCRASQSVSSAVAWYQQKPGKAPKLLIYSASSLY
SGVPSRFSGSRSGTDFTLTISSLQPEDFATYYCQQEFFVPFTFGQGTKVEIKRTVAAPSVFIFPPSDSQ
LKSGTASVVCLLNNFYPREAKVQWKVDNALQSGNSQESVTEQDSKDSTYSLSSTLTLSKADYEKHKVYA
CEVTHQGLSSPVTKSFNRGECGGSSGSGSGSTGTSSSGTGTSAGTTGTSASTSGSGSGGGGGSGGGGSA
GGTATAGASSGSEVQLVESGGGLVQPGGSLRLSCAASGFNFSSYSIHWVRQAPGKGLEWVASISPYYGS
TYYADSVKGRFTISADTSKNTAYLQMNSLRAEDTAVYYCARYKYYMSGRYTWALDYWGQGTLVTVSSAS
TKGPSVFPLAPSSKSTSGGTAALGCLVKDYFPEPVTVSWNSGALTSGVHTFPAVLQSSGLYSLSSVVTV
PSSSLGTQTYICNVNHKPSNTKVDKKVEPKSCDKTHTGGSGSAGGLNDIFEAQKIEWHEEIKRAAAINY
PYDVPDYAS

EGFP-NLS: c-Myc-NLS—EGFP (Vector backbone: pcDNA3.1)

MPAAKRVKLDLEMVSKGEELFTGVVPILVELDGDVNGHKFSVSGEGEGDATYGKLTLKFICTTGKLPVP
WPTLVTTLTYGVQCFSRYPDHMKQHDFFKSAMPEGYVQERTIFFKDDGNYKTRAEVKFEGDTLVNRIEL
KGIDFKEDGNILGHKLEYNYNSHNVYIMADKQKNGIKVNFKIRHNIEDGSVQLADHYQQNTPIGDGPVL
LPDNHYLSTQSKLSKDPNEKRDHMVLLEFVTAAGITLGMDELYK

pHTN-p97-NES: HaloTag—p97—NES—HA-Tag (Vector backbone: pHTN HaloTag CMV-neo)

MAEIGTGFPFDPHYVEVLGERMHYVDVGPRDGTPVLFLHGNPTSSYVWRNIIPHVAPTHRCIAPDLIGM
GKSDKPDLGYFFDDHVRFMDAFIEALGLEEVVLVIHDWGSALGFHWAKRNPERVKGIAFMEFIRPIPTW
DEWPEFARETFQAFRTTDVGRKLIIDQNVFIEGTLPMGVVRPLTEVEMDHYREPFLNPVDREPLWRFPN
ELPIAGEPANIVALVEEYMDWLHQSPVPKLLFWGTPGVLIPPAEAARLAKSLPNCKAVDIGPGLNLLQE
DNPDLIGSEIARWLSTLEISGEPTTEDLYFQSDNAIAMASGADSKGDDLSTAILKQKNRPNRLIVDEAI
NEDNSVVSLSQPKMDELQLFRGDTVLLKGKKRREAVCIVLSDDTCSDEKIRMNRVVRNNLRVRLGDVIS
IQPCPDVKYGKRIHVLPIDDTVEGITGNLFEVYLKPYFLEAYRPIRKGDIFLVRGGMRAVEFKVVETDP
SPYCIVAPDTVIHCEGEPIKREDEEESLNEVGYDDIGGCRKQLAQIKEMVELPLRHPALFKAIGVKPPR
GILLYGPPGTGKTLIARAVANETGAFFFLINGPEIMSKLAGESESNLRKAFEEAEKNAPAIIFIDELDA
IAPKREKTHGEVERRIVSQLLTLMDGLKQRAHVIVMAATNRPNSIDPALRRFGRFDREVDIGIPDATGR
LEILQIHTKNMKLADDVDLEQVANETHGHVGADLAALCSEAALQAIRKKMDLIDLEDETIDAEVMNSLA
VTMDDFRWALSQSNPSALRETVVEVPQVTWEDIGGLEDVKRELQELVQYPVEHPDKFLKFGMTPSKGVL
FYGPPGCGKTLLAKAIANECQANFISIKGPELLTMWFGESEANVREIFDKARQAAPCVLFFDELDSIAK
ARGGNIGDGGGAADRVINQILTEMDGMSTKKNVFIIGATNRPDIIDPAILRPGRLDQLIYIPLPDEKSR
VAILKANLRKSPVAKDVDLEFLAKMTNGFSGADLTEICQRACKLAIRESIESEIRRERERQTNPSAMEV
EEDDPVPEIRRDHFEEAMRFARRSVSDNDIRKYEMFAQTLQQSRGFGSFRFPSGNQGGAGPSQGSGGGT
GGSVYTEDNDDDLYGGSSELQNKLEELDLDSYKINYPYDVPDYAS

pHTC-p97-NES: p97—NES—HA-Tag—HaloTag (Vector backbone: pHTC HaloTag CMV-neo)

MASGADSKGDDLSTAILKQKNRPNRLIVDEAINEDNSVVSLSQPKMDELQLFRGDTVLLKGKKRREAVC
IVLSDDTCSDEKIRMNRVVRNNLRVRLGDVISIQPCPDVKYGKRIHVLPIDDTVEGITGNLFEVYLKPY
FLEAYRPIRKGDIFLVRGGMRAVEFKVVETDPSPYCIVAPDTVIHCEGEPIKREDEEESLNEVGYDDIG
GCRKQLAQIKEMVELPLRHPALFKAIGVKPPRGILLYGPPGTGKTLIARAVANETGAFFFLINGPEIMS
KLAGESESNLRKAFEEAEKNAPAIIFIDELDAIAPKREKTHGEVERRIVSQLLTLMDGLKQRAHVIVMA
ATNRPNSIDPALRRFGRFDREVDIGIPDATGRLEILQIHTKNMKLADDVDLEQVANETHGHVGADLAAL
CSEAALQAIRKKMDLIDLEDETIDAEVMNSLAVTMDDFRWALSQSNPSALRETVVEVPQVTWEDIGGLE
DVKRELQELVQYPVEHPDKFLKFGMTPSKGVLFYGPPGCGKTLLAKAIANECQANFISIKGPELLTMWF
GESEANVREIFDKARQAAPCVLFFDELDSIAKARGGNIGDGGGAADRVINQILTEMDGMSTKKNVFIIG
ATNRPDIIDPAILRPGRLDQLIYIPLPDEKSRVAILKANLRKSPVAKDVDLEFLAKMTNGFSGADLTEI
CQRACKLAIRESIESEIRRERERQTNPSAMEVEEDDPVPEIRRDHFEEAMRFARRSVSDNDIRKYEMFA
QTLQQSRGFGSFRFPSGNQGGAGPSQGSGGGTGGSVYTEDNDDDLYGGSSELQNKLEELDLDSYKINYP
YDVPDYAEPTTEDLYFQSDNDGSEIGTGFPFDPHYVEVLGERMHYVDVGPRDGTPVLFLHGNPTSSYVW
RNIIPHVAPTHRCIAPDLIGMGKSDKPDLGYFFDDHVRFMDAFIEALGLEEVVLVIHDWGSALGFHWAK
RNPERVKGIAFMEFIRPIPTWDEWPEFARETFQAFRTTDVGRKLIIDQNVFIEGTLPMGVVRPLTEVEM
DHYREPFLNPVDREPLWRFPNELPIAGEPANIVALVEEYMDWLHQSPVPKLLFWGTPGVLIPPAEAARL
AKSLPNCKAVDIGPGLNLLQEDNPDLIGSEIARWLSTLEISG

pNLF1N-p97-NES: NanoLuc—p97—NES—HA-Tag (Vector backbone: pNLF1-N CMV/Hygro)

MVFTLEDFVGDWRQTAGYNLDQVLEQGGVSSLFQNLGVSVTPIQRIVLSGENGLKIDIHVIIPYEGLSG
DQMGQIEKIFKVVYPVDDHHFKVILHYGTLVIDGVTPNMIDYFGRPYEGIAVFDGKKITVTGTLWNGNK
IIDERLINPDGSLLFRVTINGVTGWRLCERILAGSSGAIAMASGADSKGDDLSTAILKQKNRPNRLIVD
EAINEDNSVVSLSQPKMDELQLFRGDTVLLKGKKRREAVCIVLSDDTCSDEKIRMNRVVRNNLRVRLGD
VISIQPCPDVKYGKRIHVLPIDDTVEGITGNLFEVYLKPYFLEAYRPIRKGDIFLVRGGMRAVEFKVVE

TDPSPYCIVAPDTVIHCEGEPIKREDEEESLNEVGYDDIGGCRKQLAQIKEMVELPLRHPALFKAIGVK
PPRGILLYGPPGTGKTLIARAVANETGAFFFLINGPEIMSKLAGESESNLRKAFEEAEKNAPAIIFIDE
LDAIAPKREKTHGEVERRIVSQLLTLMDGLKQRAHVIVMAATNRPNSIDPALRRFGRFDREVDIGIPDA
TGRLEILQIHTKNMKLADDVDLEQVANETHGHVGADLAALCSEAALQAIRKKMDLIDLEDETIDAEVMN
SLAVTMDDFRWALSQSNPSALRETVVEVPQVTWEDIGGLEDVKRELQELVQYPVEHPDKFLKFGMTPSK
GVLFYGPPGCGKTLLAKAIANECQANFISIKGPELLTMWFGESEANVREIFDKARQAAPCVLFFDELDS
IAKARGGNIGDGGGAADRVINQILTEMDGMSTKKNVFIIGATNRPDIIDPAILRPGRLDQLIYIPLPDE
KSRVAILKANLRKSPVAKDVDLEFLAKMTNGFSGADLTEICQRACKLAIRESIESEIRRERERQTNPSA
MEVEEDDPVPEIRRDHFEEAMRFARRSVSDNDIRKYEMFAQTLQQSRGFGSFRFPSGNQGGAGPSQGSG
GGTGGSVYTEDNDDDLYGGSSELQNKLEELDLDSYKINYPYDVPDYAS

pNLF1C-p97-NES: p97—NES—HA-Tag—NanoLuc (Vector backbone: pNLF1-C CMV/Hygro)

MASGADSKGDDLSTAILKQKNRPNRLIVDEAINEDNSVVSLSQPKMDELQLFRGDTVLLKGKKRREAVC
IVLSDDTCSDEKIRMNRVVRNNLRVRLGDVISIQPCPDVKYGKRIHVLPIDDTVEGITGNLFEVYLKPY
FLEAYRPIRKGDIFLVRGGMRAVEFKVVETDPSPYCIVAPDTVIHCEGEPIKREDEEESLNEVGYDDIG
GCRKQLAQIKEMVELPLRHPALFKAIGVKPPRGILLYGPPGTGKTLIARAVANETGAFFFLINGPEIMS
KLAGESESNLRKAFEEAEKNAPAIIFIDELDAIAPKREKTHGEVERRIVSQLLTLMDGLKQRAHVIVMA
ATNRPNSIDPALRRFGRFDREVDIGIPDATGRLEILQIHTKNMKLADDVDLEQVANETHGHVGADLAAL
CSEAALQAIRKKMDLIDLEDETIDAEVMNSLAVTMDDFRWALSQSNPSALRETVVEVPQVTWEDIGGLE
DVKRELQELVQYPVEHPDKFLKFGMTPSKGVLFYGPPGCGKTLLAKAIANECQANFISIKGPELLTMWF
GESEANVREIFDKARQAAPCVLFFDELDSIAKARGGNIGDGGGAADRVINQILTEMDGMSTKKNVFIIG
ATNRPDIIDPAILRPGRLDQLIYIPLPDEKSRVAILKANLRKSPVAKDVDLEFLAKMTNGFSGADLTEI
CQRACKLAIRESIESEIRRERERQTNPSAMEVEEDDPVPEIRRDHFEEAMRFARRSVSDNDIRKYEMFA
QTLQQSRGFGSFRFPSGNQGGAGPSQGSGGGTGGSVYTEDNDDDLYGGSSELQNKLEELDLDSYKINYP
YDVPDYAGSSGVFTLEDFVGDWRQTAGYNLDQVLEQGGVSSLFQNLGVSVTPIQRIVLSGENGLKIDIH
VIIPYEGLSGDQMGQIEKIFKVVYPVDDHHFKVILHYGTLVIDGVTPNMIDYFGRPYEGIAVFDGKKIT
VTGTLWNGNKIIDERLINPDGSLLFRVTINGVTGWRLCERILA

pHTN-p47-NES: HaloTag—p47—NES—FLAG-Tag (Vector backbone: pHTN HaloTag CMV-neo)

MAEIGTGFPFDPHYVEVLGERMHYVDVGPRDGTPVLFLHGNPTSSYVWRNIIPHVAPTHRCIAPDLIGM
GKSDKPDLGYFFDDHVRFMDAFIEALGLEEVVLVIHDWGSALGFHWAKRNPERVKGIAFMEFIRPIPTW
DEWPEFARETFQAFRTTDVGRKLIIDQNVFIEGTLPMGVVRPLTEVEMDHYREPFLNPVDREPLWRFPN
ELPIAGEPANIVALVEEYMDWLHQSPVPKLLFWGTPGVLIPPAEAARLAKSLPNCKAVDIGPGLNLLQE
DNPDLIGSEIARWLSTLEISGEPTTEDLYFQSDNAIAMAAERQEALREFVAVTGAEEDRARFFLESAGW
DLQIALASFYEDGGDEDIVTISQATPSSVSRGTAPSDNRVTSFRDLIHDQDEDEEEEEGQRFYAGGSER
SGQQIVGPPRKKSPNELVDDLFKGAKEHGAVAVERVTKSPGETSKPRPFAGGGYRLGAAPEEESAYVAG
EKRQHSSQDVHVVLKLWKSGFSLDNGELRSYQDPSNAQFLESIRRGEVPAELRRLAHGGQVNLDMEDHR
DEDFVKPKGAFKAFTGEGQKLGSTAPQVLSTSSPAQQAENEAKASSSILIDESEPTTNIQIRLADGGRL
VQKFNHSHRISDIRLFIVDARPAMAATSFILMTTFPNKELADESQTLKEANLLNAVIVQRLTGSSELQN
KLEELDLDSYKINDYKDDDDK

pHTC-p47-NES: p47—NES—FLAG-Tag—HaloTag (Vector backbone: pHTC HaloTag CMV-neo)

MAAERQEALREFVAVTGAEEDRARFFLESAGWDLQIALASFYEDGGDEDIVTISQATPSSVSRGTAPSD
NRVTSFRDLIHDQDEDEEEEEGQRFYAGGSERSGQQIVGPPRKKSPNELVDDLFKGAKEHGAVAVERVT
KSPGETSKPRPFAGGGYRLGAAPEEESAYVAGEKRQHSSQDVHVVLKLWKSGFSLDNGELRSYQDPSNA
QFLESIRRGEVPAELRRLAHGGQVNLDMEDHRDEDFVKPKGAFKAFTGEGQKLGSTAPQVLSTSSPAQQ
AENEAKASSSILIDESEPTTNIQIRLADGGRLVQKFNHSHRISDIRLFIVDARPAMAATSFILMTTFPN
KELADESQTLKEANLLNAVIVQRLTGSSELQNKLEELDLDSYKINDYKDDDDKEPTTEDLYFQSDNDGS
EIGTGFPFDPHYVEVLGERMHYVDVGPRDGTPVLFLHGNPTSSYVWRNIIPHVAPTHRCIAPDLIGMGK
SDKPDLGYFFDDHVRFMDAFIEALGLEEVVLVIHDWGSALGFHWAKRNPERVKGIAFMEFIRPIPTWDE
WPEFARETFQAFRTTDVGRKLIIDQNVFIEGTLPMGVVRPLTEVEMDHYREPFLNPVDREPLWRFPNEL
PIAGEPANIVALVEEYMDWLHQSPVPKLLFWGTPGVLIPPAEAARLAKSLPNCKAVDIGPGLNLLQEDN
PDLIGSEIARWLSTLEISG

pNLF1N-p47-NES: NanoLuc—p47—NES—FLAG-Tag (Vector backbone: pNLF1-N CMV/Hygro)

MVFTLEDFVGDWRQTAGYNLDQVLEQGGVSSLFQNLGVSVTPIQRIVLSGENGLKIDIHVIIPYEGLSG
DQMGQIEKIFKVVYPVDDHHFKVILHYGTLVIDGVTPNMIDYFGRPYEGIAVFDGKKITVTGTLWNGNK
IIDERLINPDGSLLFRVTINGVTGWRLCERILAGSSGAIASEFRAQMAAERQEALREFVAVTGAEEDRA
RFFLESAGWDLQIALASFYEDGGDEDIVTISQATPSSVSRGTAPSDNRVTSFRDLIHDQDEDEEEEEGQ
RFYAGGSERSGQQIVGPPRKKSPNELVDDLFKGAKEHGAVAVERVTKSPGETSKPRPFAGGGYRLGAAP
EEESAYVAGEKRQHSSQDVHVVLKLWKSGFSLDNGELRSYQDPSNAQFLESIRRGEVPAELRRLAHGGQ
VNLDMEDHRDEDFVKPKGAFKAFTGEGQKLGSTAPQVLSTSSPAQQAENEAKASSSILIDESEPTTNIQ
IRLADGGRLVQKFNHSHRISDIRLFIVDARPAMAATSFILMTTFPNKELADESQTLKEANLLNAVIVQR
LTGSSELQNKLEELDLDSYKINDYKDDDDK

pNLF1C-p47-NES: p47—NES—FLAG-Tag—NanoLuc (Vector backbone: pNLF1-C CMV/Hygro)

MAAERQEALREFVAVTGAEEDRARFFLESAGWDLQIALASFYEDGGDEDIVTISQATPSSVSRGTAPSD
NRVTSFRDLIHDQDEDEEEEEGQRFYAGGSERSGQQIVGPPRKKSPNELVDDLFKGAKEHGAVAVERVT
KSPGETSKPRPFAGGGYRLGAAPEEESAYVAGEKRQHSSQDVHVVLKLWKSGFSLDNGELRSYQDPSNA
QFLESIRRGEVPAELRRLAHGGQVNLDMEDHRDEDFVKPKGAFKAFTGEGQKLGSTAPQVLSTSSPAQQ
AENEAKASSSILIDESEPTTNIQIRLADGGRLVQKFNHSHRISDIRLFIVDARPAMAATSFILMTTFPN
KELADESQTLKEANLLNAVIVQRLTGSSELQNKLEELDLDSYKINDYKDDDDKGSSGVFTLEDFVGDWR
QTAGYNLDQVLEQGGVSSLFQNLGVSVTPIQRIVLSGENGLKIDIHVIIPYEGLSGDQMGQIEKIFKVV
YPVDDHHFKVILHYGTLVIDGVTPNMIDYFGRPYEGIAVFDGKKITVTGTLWNGNKIIDERLINPDGSL
LFRVTINGVTGWRLCERILA

pNLF1N-p97: NanoLuc—p97—HA-Tag (Vector backbone: pNLF1-N CMV/Hygro)

MVFTLEDFVGDWRQTAGYNLDQVLEQGGVSSLFQNLGVSVTPIQRIVLSGENGLKIDIHVIIPYEGLSG
DQMGQIEKIFKVVYPVDDHHFKVILHYGTLVIDGVTPNMIDYFGRPYEGIAVFDGKKITVTGTLWNGNK
IIDERLINPDGSLLFRVTINGVTGWRLCERILAGSSGAIAMASGADSKGDDLSTAILKQKNRPNRLIVD
EAINEDNSVVSLSQPKMDELQLFRGDTVLLKGKKRREAVCIVLSDDTCSDEKIRMNRVVRNNLRVRLGD
VISIQPCPDVKYGKRIHVLPIDDTVEGITGNLFEVYLKPYFLEAYRPIRKGDIFLVRGGMRAVEFKVVE
TDPSPYCIVAPDTVIHCEGEPIKREDEEESLNEVGYDDIGGCRKQLAQIKEMVELPLRHPALFKAIGVK

PPRGILLYGPPGTGKTLIARAVANETGAFFFLINGPEIMSKLAGESESNLRKAFEEAEKNAPAIIFIDE
LDAIAPKREKTHGEVERRIVSQLLTLMDGLKQRAHVIVMAATNRPNSIDPALRRFGRFDREVDIGIPDA
TGRLEILQIHTKNMKLADDVDLEQVANETHGHVGADLAALCSEAALQAIRKKMDLIDLEDETIDAEVMN
SLAVTMDDFRWALSQSNPSALRETVVEVPQVTWEDIGGLEDVKRELQELVQYPVEHPDKFLKFGMTPSK
GVLFYGPPGCGKTLLAKAIANECQANFISIKGPELLTMWFGESEANVREIFDKARQAAPCVLFFDELDS
IAKARGGNIGDGGGAADRVINQILTEMDGMSTKKNVFIIGATNRPDIIDPAILRPGRLDQLIYIPLPDE
KSRVAILKANLRKSPVAKDVDLEFLAKMTNGFSGADLTEICQRACKLAIRESIESEIRRERERQTNPSA
MEVEEDDPVPEIRRDHFEEAMRFARRSVSDNDIRKYEMFAQTLQQSRGFGSFRFPSGNQGGAGPSQGSG
GGTGGSVYTEDNDDDLYGINYPYDVPDYAS

pHTC-p47: p47—FLAG-Tag—HaloTag (Vector backbone: pHTC HaloTag CMV-neo)

MAAERQEALREFVAVTGAEEDRARFFLESAGWDLQIALASFYEDGGDEDIVTISQATPSSVSRGTAPSD
NRVTSFRDLIHDQDEDEEEEEGQRFYAGGSERSGQQIVGPPRKKSPNELVDDLFKGAKEHGAVAVERVT
KSPGETSKPRPFAGGGYRLGAAPEEESAYVAGEKRQHSSQDVHVVLKLWKSGFSLDNGELRSYQDPSNA
QFLESIRRGEVPAELRRLAHGGQVNLDMEDHRDEDFVKPKGAFKAFTGEGQKLGSTAPQVLSTSSPAQQ
AENEAKASSSILIDESEPTTNIQIRLADGGRLVQKFNHSHRISDIRLFIVDARPAMAATSFILMTTFPN
KELADESQTLKEANLLNAVIVQRLTINDYKDDDDKEPTTEDLYFQSDNDGSEIGTGFPFDPHYVEVLGE
RMHYVDVGPRDGTPVLFLHGNPTSSYVWRNIIPHVAPTHRCIAPDLIGMGKSDKPDLGYFFDDHVRFMD
AFIEALGLEEVVLVIHDWGSALGFHWAKRNPERVKGIAFMEFIRPIPTWDEWPEFARETFQAFRTTDVG
RKLIIDQNVFIEGTLPMGVVRPLTEVEMDHYREPFLNPVDREPLWRFPNELPIAGEPANIVALVEEYMD
WLHQSPVPKLLFWGTPGVLIPPAEAARLAKSLPNCKAVDIGPGLNLLQEDNPDLIGSEIARWLSTLEIS
G

pNLF1N-p97-N: NanoLuc—p97-N—HA-Tag (Vector backbone: pNLF1-N CMV/Hygro)

MVFTLEDFVGDWRQTAGYNLDQVLEQGGVSSLFQNLGVSVTPIQRIVLSGENGLKIDIHVIIPYEGLSG
DQMGQIEKIFKVVYPVDDHHFKVILHYGTLVIDGVTPNMIDYFGRPYEGIAVFDGKKITVTGTLWNGNK
IIDERLINPDGSLLFRVTINGVTGWRLCERILAGSSGAIAMASGADSKGDDLSTAILKQKNRPNRLIVD
EAINEDNSVVSLSQPKMDELQLFRGDTVLLKGKKRREAVCIVLSDDTCSDEKIRMNRVVRNNLRVRLGD
VISIQPCPDVKYGKRIHVLPIDDTVEGITGNLFEVYLKPYFLEAYRPIRKGDIFLVRGGMRAVEFKVVE
TDPSPYCIVAPDTVIHCEGEINYPYDVPDYAS

pHTC-p47-UBX: p47-UBX—FLAG-Tag—HaloTag (Vector backbone: pHTC HaloTag CMV-neo)

MVKPKGAFKAFTGEGQKLGSTAPQVLSTSSPAQQAENEAKASSSILIDESEPTTNIQIRLADGGRLVQK
FNHSHRISDIRLFIVDARPAMAATSFILMTTFPNKELADESQTLKEANLLNAVIVQRLTINDYKDDDDK
EPTTEDLYFQSDNDGSEIGTGFPFDPHYVEVLGERMHYVDVGPRDGTPVLFLHGNPTSSYVWRNIIPHV
APTHRCIAPDLIGMGKSDKPDLGYFFDDHVRFMDAFIEALGLEEVVLVIHDWGSALGFHWAKRNPERVK
GIAFMEFIRPIPTWDEWPEFARETFQAFRTTDVGRKLIIDQNVFIEGTLPMGVVRPLTEVEMDHYREPF
LNPVDREPLWRFPNELPIAGEPANIVALVEEYMDWLHQSPVPKLLFWGTPGVLIPPAEAARLAKSLPNC
KAVDIGPGLNLLQEDNPDLIGSEIARWLSTLEISG

p47-FLAG: FLAG-Tag—p47 (Vector backbone: pcDNA3.1)

MDYKDDDDKGGMAAERQEALREFVAVTGAEEDRARFFLESAGWDLQIALASFYEDGGDEDIVTISQATP
SSVSRGTAPSDNRVTSFRDLIHDQDEDEEEEEGQRFYAGGSERSGQQIVGPPRKKSPNELVDDLFKGAK
EHGAVAVERVTKSPGETSKPRPFAGGGYRLGAAPEEESAYVAGEKRQHSSQDVHVVLKLWKSGFSLDNG
ELRSYQDPSNAQFLESIRRGEVPAELRRLAHGGQVNLDMEDHRDEDFVKPKGAFKAFTGEGQKLGSTAP
QVLSTSSPAQQAENEAKASSSILIDESEPTTNIQIRLADGGRLVQKFNHSHRISDIRLFIVDARPAMAA
TSFILMTTFPNKELADESQTLKEANLLNAVIVQRLT

4. Spectral and characterization data

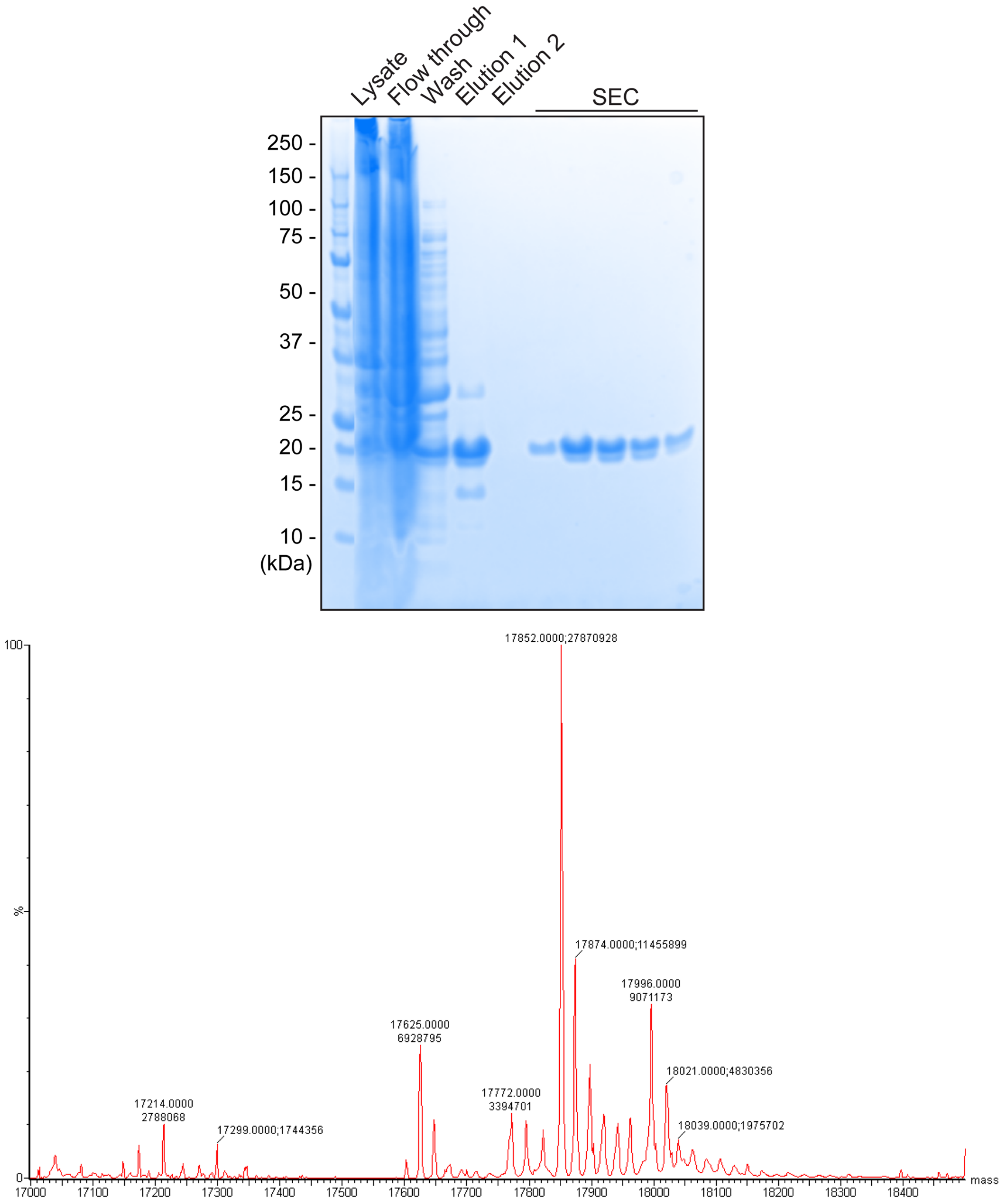

Figure SP1. SDS-PAGE analysis and representative LC-MS spectrum of p47-UBX after biotinylation. Note that the minor existence of unbiotinylated proteins does not affect the phage display process; the irrelevant lanes between the ladder lane and the "Lysate" lane on the same gel were removed for clear visualizations.

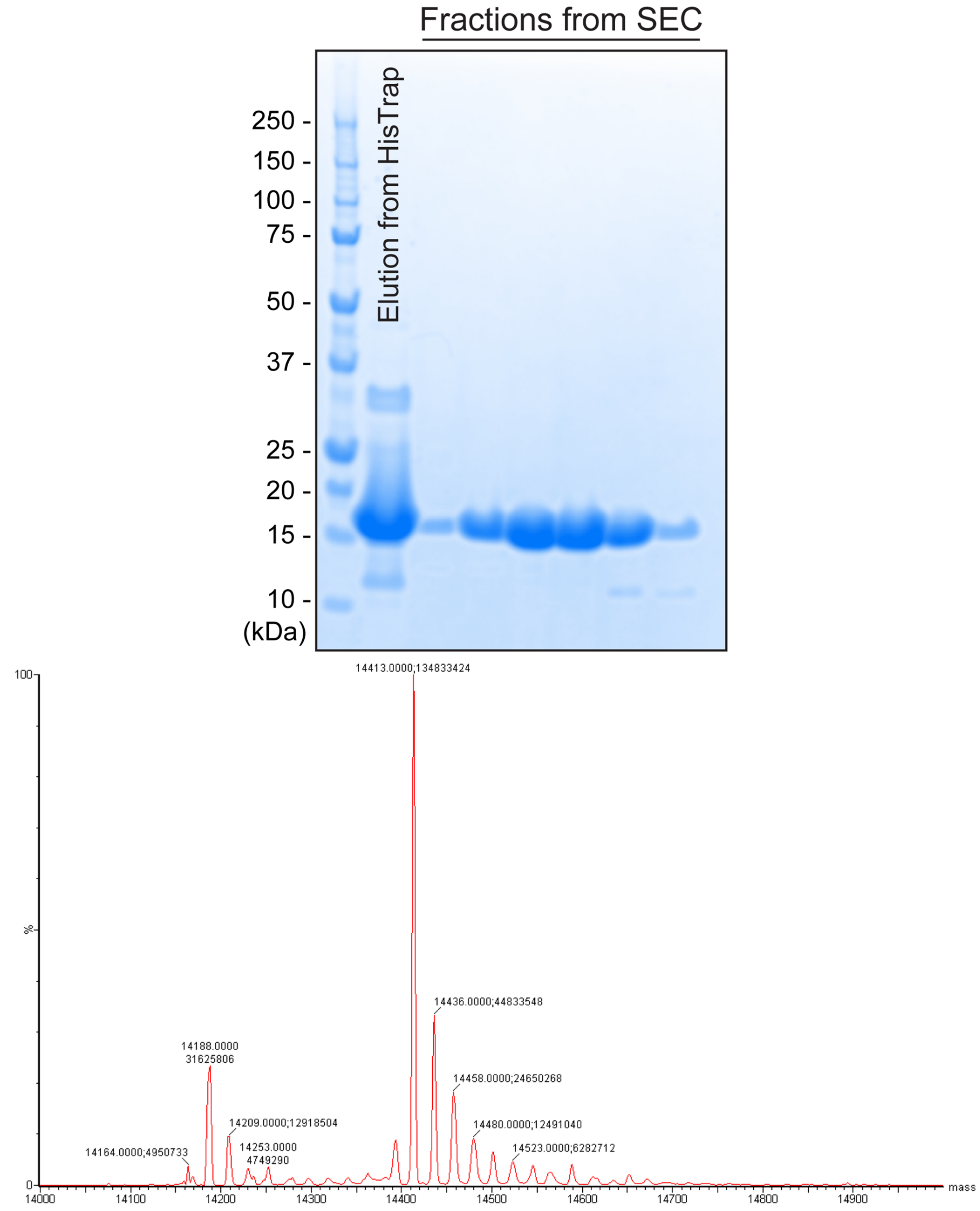

Figure SP2. SDS-PAGE analysis and representative LC-MS spectrum of NPL4-UBXL after biotinylation.

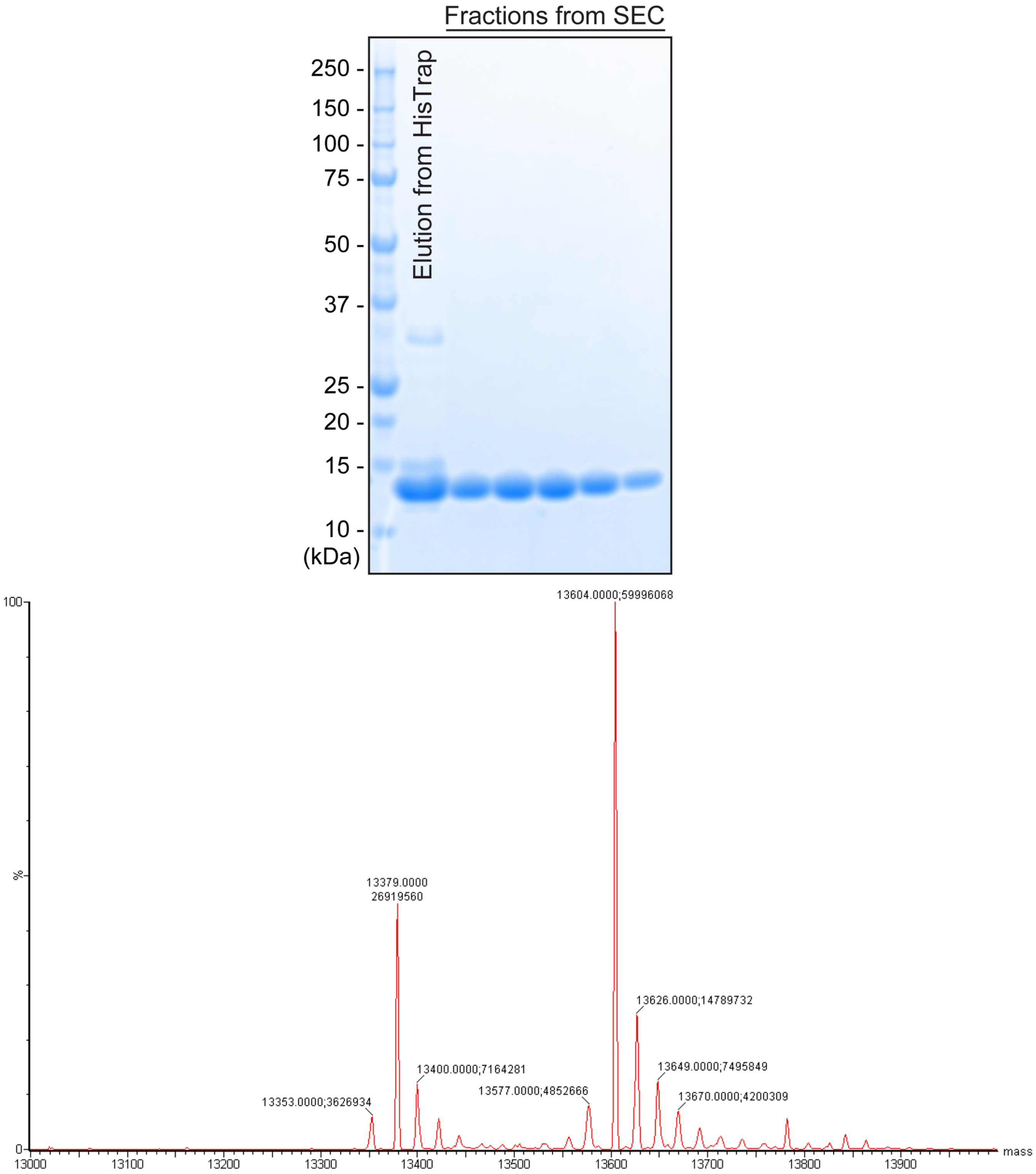

Figure SP3. SDS-PAGE analysis and representative LC-MS spectrum of FAF1-UBX after biotinylation.

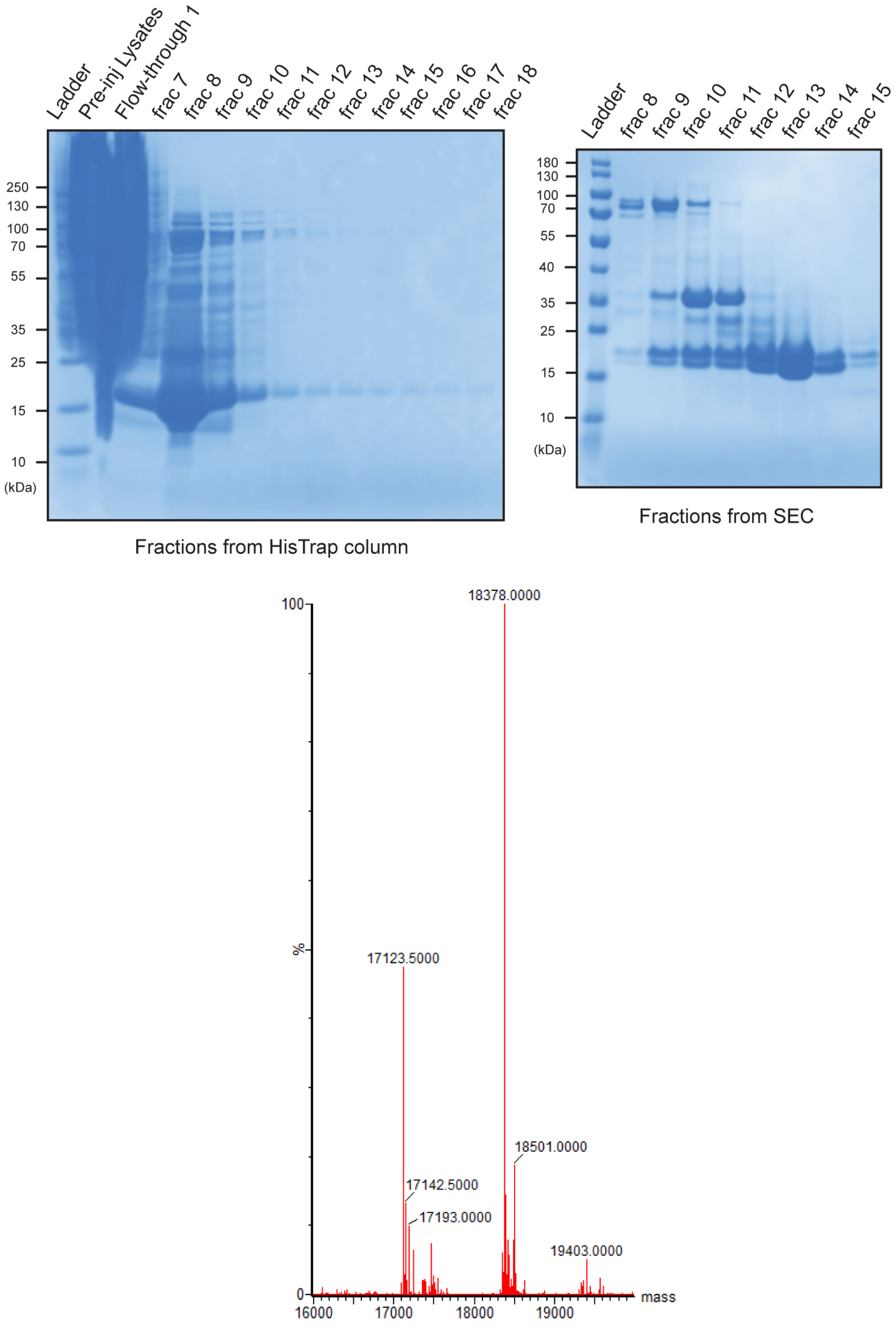

Figure SP4. SDS-PAGE analysis and representative LC-MS spectrum of p37-UBX after biotinylation.

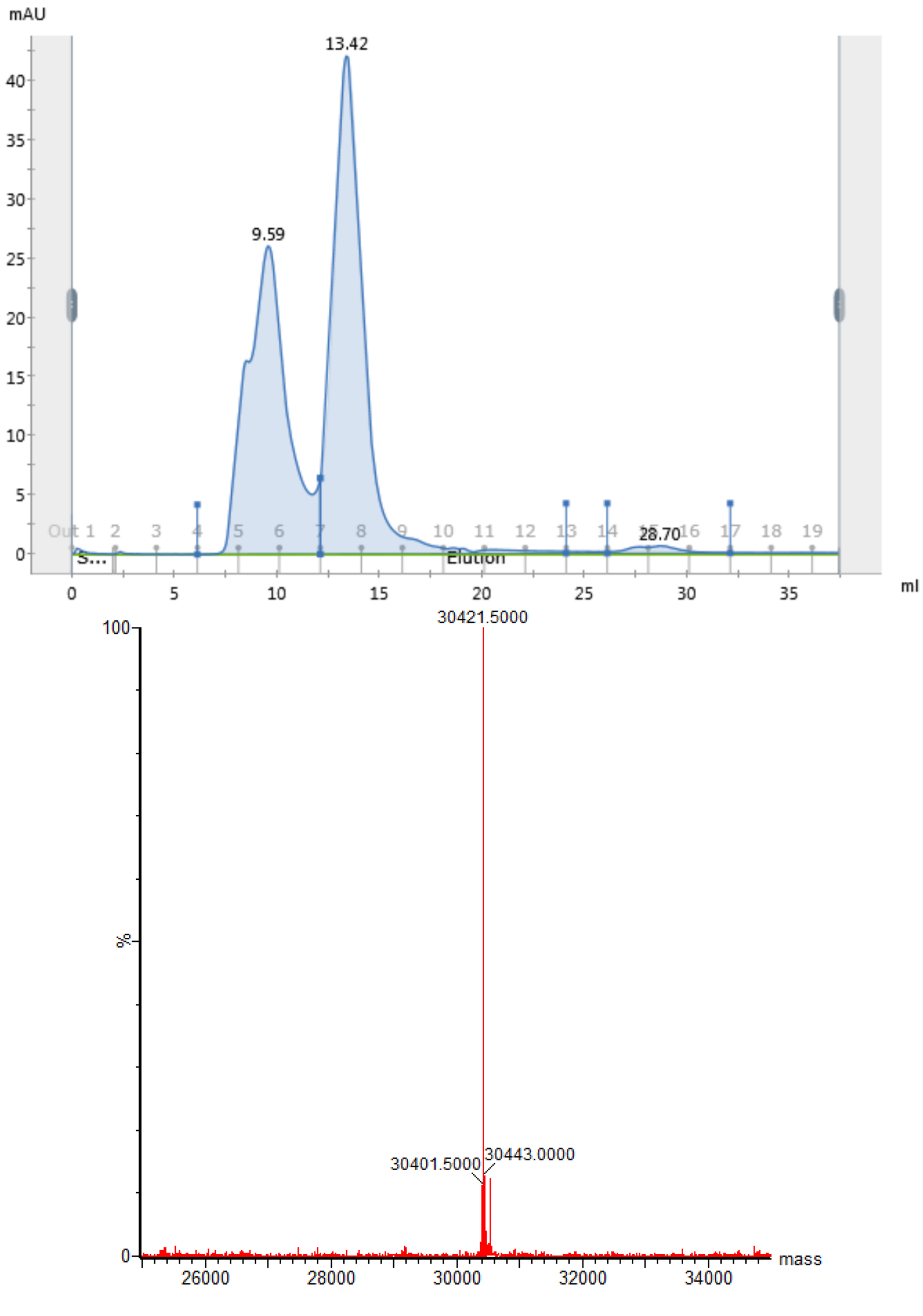

Figure SP5. Size-exclusion chromatography (SEC) curve and representative LC-MS spectrum of scFv-A04.

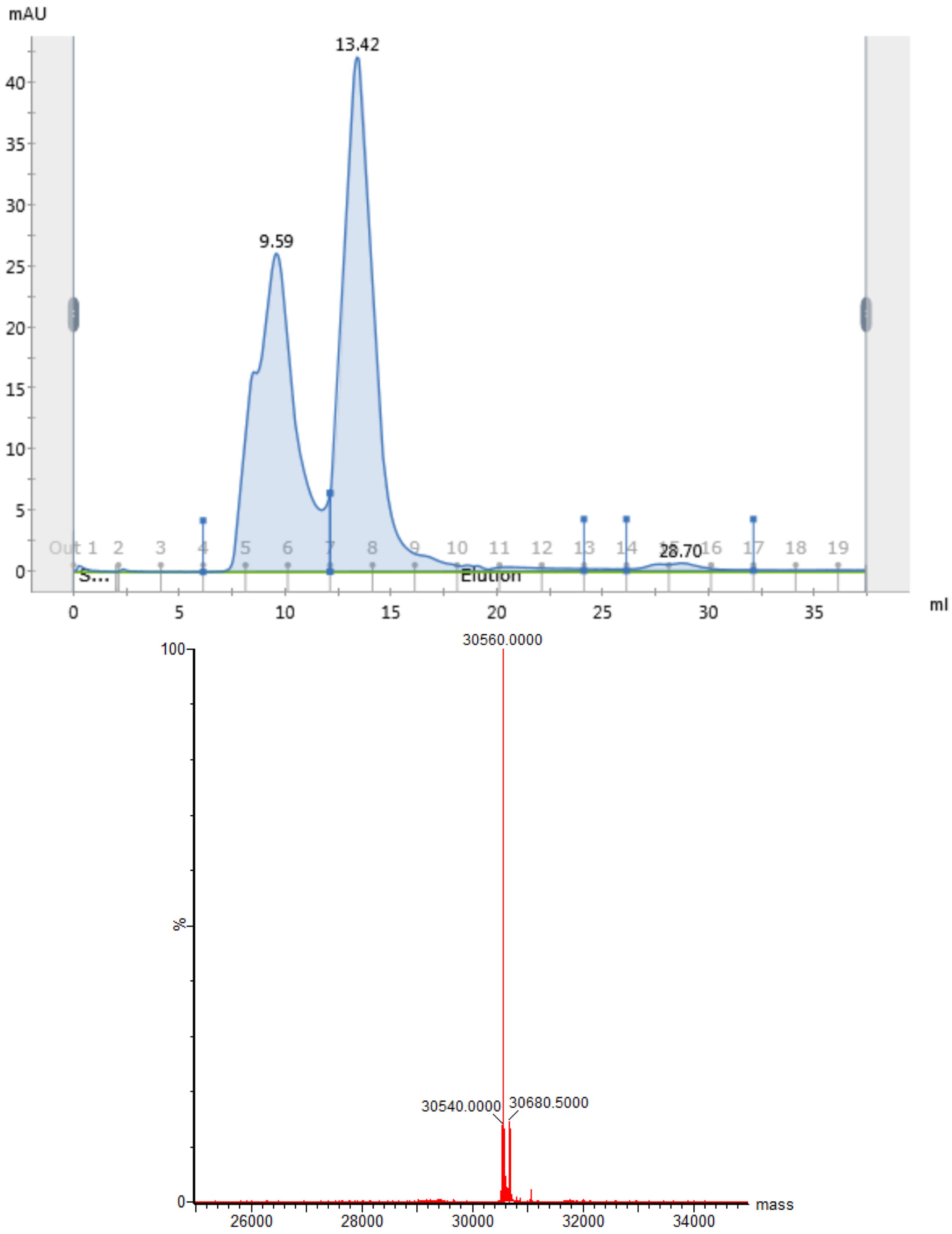

Figure SP6. SEC curve and representative LC-MS spectrum of scFv-A06.

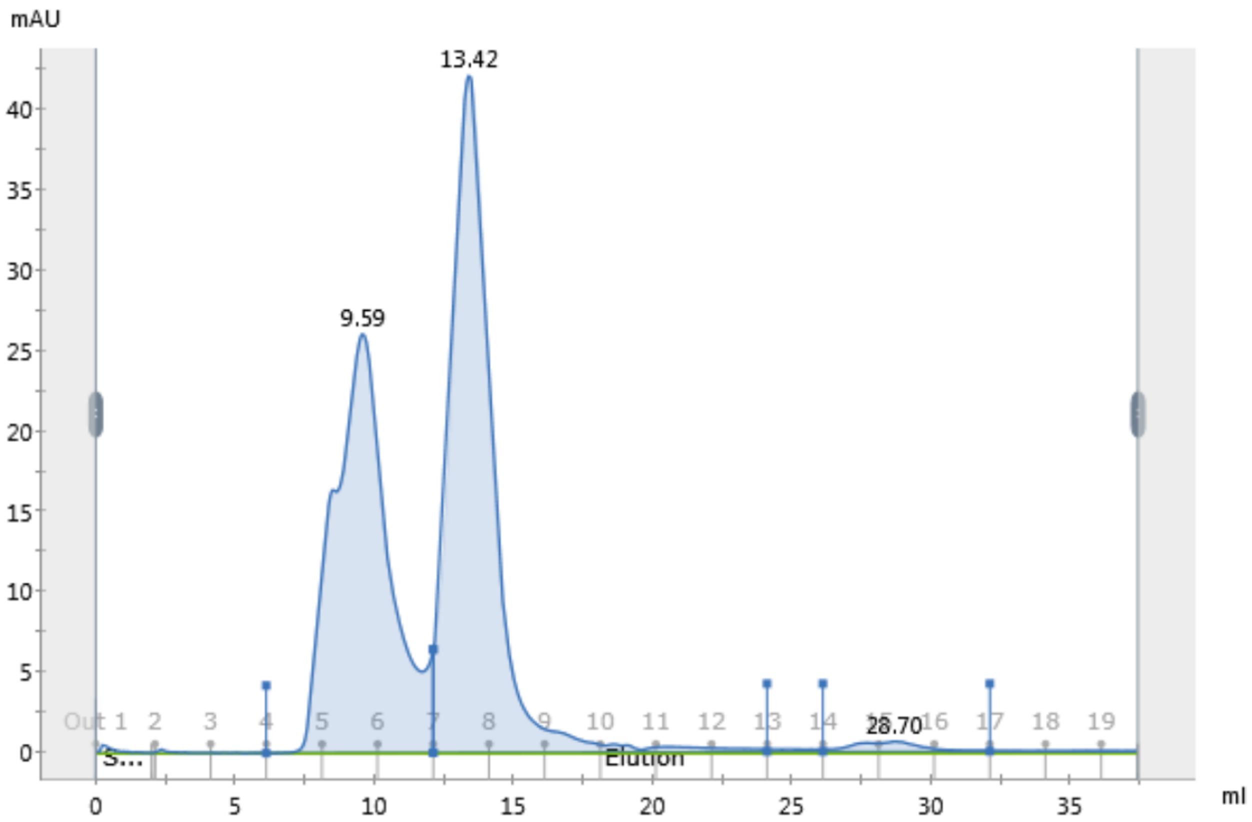

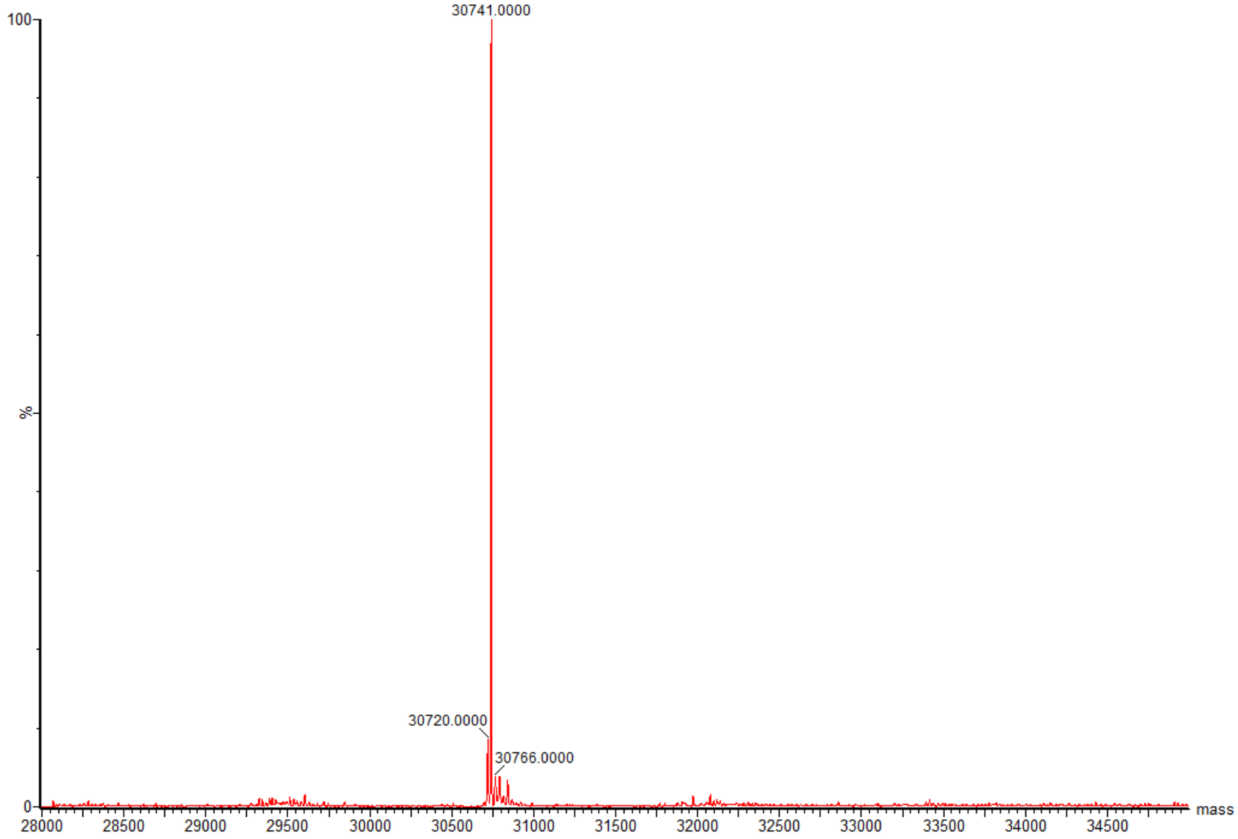

Figure SP7. SEC curve and representative LC-MS spectrum of scFv-B01.

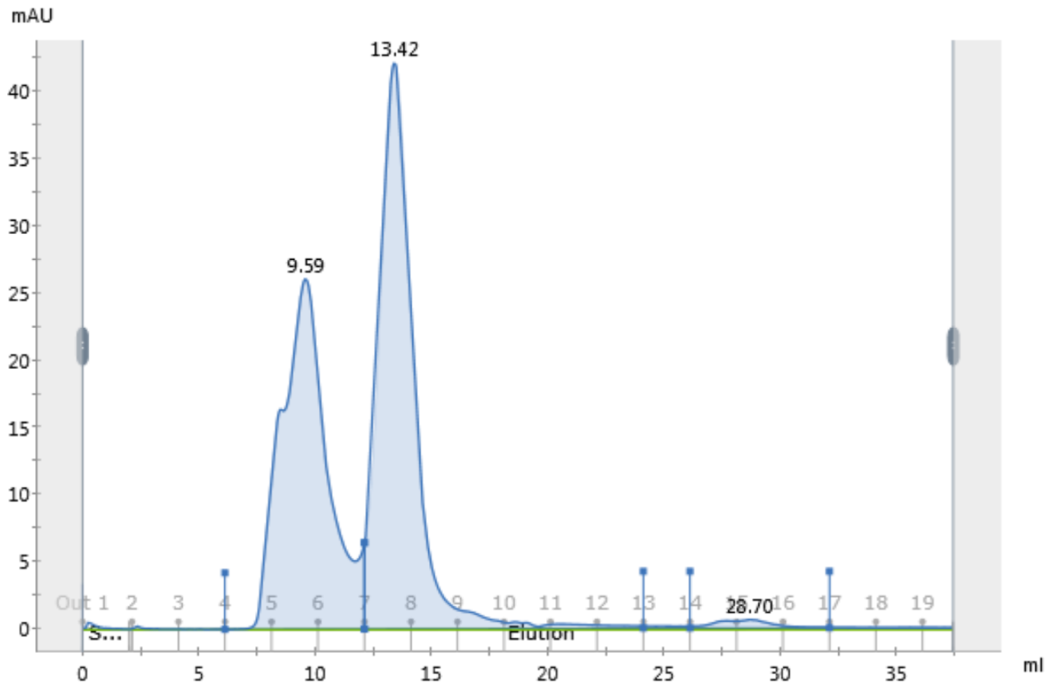

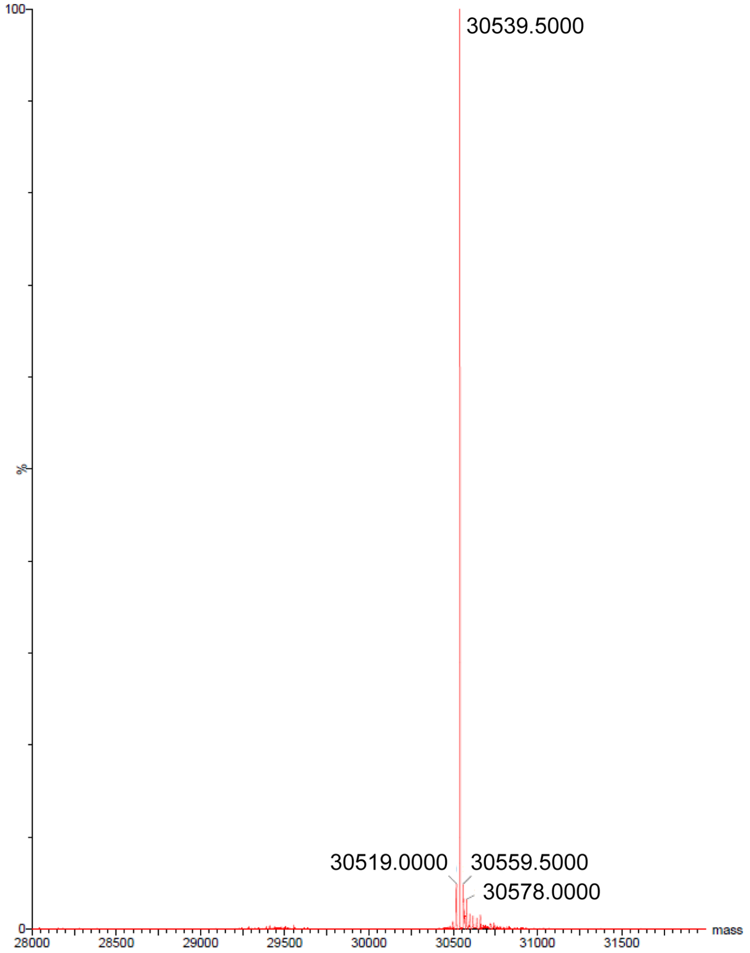

Figure SP8. SEC curve and representative LC-MS spectrum of scFv-E04.

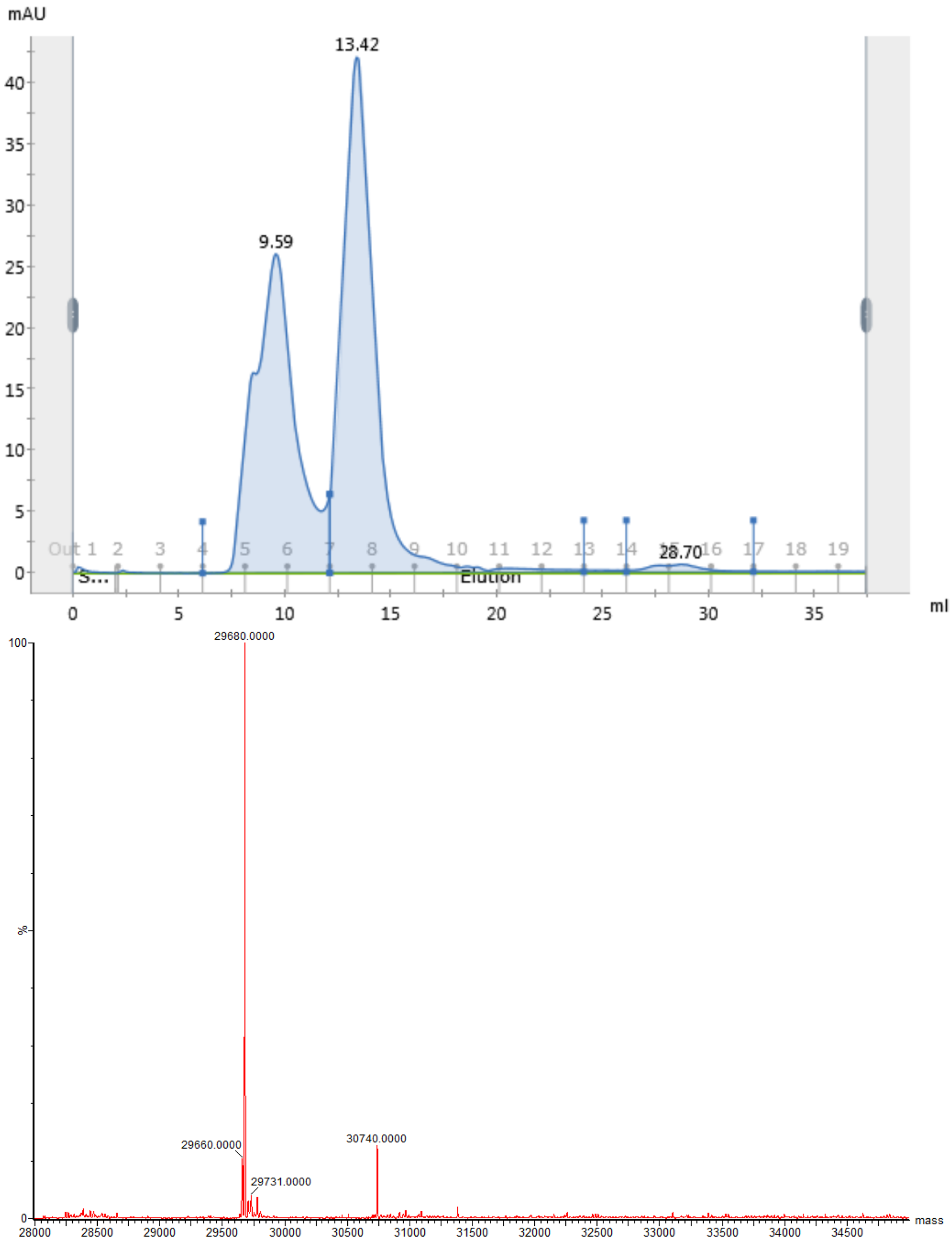

Figure SP9. SEC curve and representative LC-MS spectrum of scFv-G08.

5. References

SR1. Dreveny, I.; Kondo, H.; Uchiyama, K.; Shaw, A.; Zhang, X.; Freemont, P. S. Structural basis of the interaction between the AAA ATPase p97/VCP and its adaptor protein p47. *EMBO J.* **2004**, *23*, 1030-1039.

SR2. Xue, L.; Blythe, E. E.; Freiberger, E. C.; Mamrosh, J. L.; Hebert, A. S.; Reitsma, J. M.; Hess, S.; Coon, J. J.; Deshaies, R. J. Valosin-containing protein (VCP)-Adaptor Interactions are Exceptionally Dynamic and Subject to Differential Modulation by a VCP Inhibitor. *Mol. Cell. Proteomics* **2016**, *15*, 2970-2986.

SR3. Chou, T. F.; Bulfer, S. L.; Weihi, C. C.; Li, K. L.; Lis, L. G.; Walters, M. A.; Schoenen, F. J.; Lin, H. J.; Deshaies, R. J.; Arkin, M. R. Specific Inhibition of p97/VCP ATPase and Kinetic Analysis Demonstrate Interaction between D1 and D2 ATPase Domains. *J. Mol. Biol.* **2014**, *426*, 2886-2899.

SR4. Bulfer, S. L.; Chou, T. F.; Arkin, M. R. p97 Disease Mutations Modulate Nucleotide-Induced Conformation to Alter Protein-Protein Interactions. *ACS Chem. Biol.* **2016**, *11*, 2112-2116.

SR5. Zhou, Y.; Marks, J. D. Discovery of Internalizing Antibodies to Tumor Antigens from Phage Libraries. *Methods Enzymol.* **2012**, *502*, 43-66.

SR6. Hill, Z. B.; Martinko, A. J.; Nguyen, D. P.; Wells, J. A. Human antibody-based chemically induced dimerizers for cell therapeutic applications. *Nat. Chem. Biol.* **2018**, *14*, 112-117.

SR7. Heckman, K. L.; Pease, L. R. Gene splicing and mutagenesis by PCR-driven overlap extension. *Nat. Protoc.* **2007**, *2*, 924-932.

SR8. Ray, M.; Tang, R.; Jiang, Z. W.; Rotello, V. M. Quantitative Tracking of Protein Trafficking to the Nucleus Using Cytosolic Protein Delivery by Nanoparticle-Stabilized Nanocapsules. *Bioconjugate Chem.* **2015**, *26*, 1004-1007.

SR9. Hornsby, M.; Paduch, M.; Miersch, S.; Saaf, A.; Matsuguchi, T.; Lee, B.; Wypisniak, K.; Doak, A.; King, D.; Usatyuk, S.; Perry, K.; Lu, V.; Thomas, W.; Luke, J.; Goodman, J.; Hoey, R. J.; Lai, D.; Griffin, C.; Li, Z. J.; Vizeacoumar, F. J.; Dong, D.; Campbell, E.; Anderson, S.; Zhong, N.; Graslund, S.; Koide, S.; Moffat, J.; Sidhu, S.; Kossiakoff, A.; Wells, J. A High Through-put Platform for Recombinant Antibodies to Folded Proteins. *Mol. Cell. Proteomics* **2015**, *14*, 2833-2847.

SR10. Goedhart, J.; von Stetten, D.; Noirclerc-Savoye, M.; Lelimousin, M.; Joosen, L.; Hink, M. A.; van Weeren, L.; Gadella, T. W. J.; Royant, A. Structure-guided evolution of cyan fluorescent proteins towards a quantum yield of 93%. *Nat. Commun.* **2012**, *3*, 751.

SR11. Rabouille, C.; Levine, T. P.; Peters, J.-M.; Warren, G. An NSF-like ATPase, p97, and NSF mediate cisternal regrowth from mitotic golgi fragments. *Cell* **1995**, *82*, 905-914.

SR12. Tang, D. M.; Xiang, Y.; Wang, Y. Z. Reconstitution of the cell cycle-regulated Golgi disassembly and reassembly in a cell-free system. *Nat. Protoc.* **2010**, *5*, 758-772.

SR13. Katakura, Y.; Kobayashi, E.; Kurokawa, Y.; Omasa, T.; Fujiyama, K.; Suga, K.-I. Cloning of cDNA and characterization of anti-RNase A monoclonal antibody 3A21. *J. Ferment. Bioeng.* **1996**, *82*, 312-314.
